# The contribution of photodegradation to litter decomposition in a temperate forest gap and understorey

**DOI:** 10.1111/nph.17022

**Published:** 2020-11-30

**Authors:** Qing‐Wei Wang, Marta Pieristè, Chenggang Liu, Tanaka Kenta, Thomas Matthew Robson, Hiroko Kurokawa

**Affiliations:** ^1^ CAS Key Laboratory of Forest Ecology and Management Institute of Applied Ecology Chinese Academy of Sciences Shenyang 110016 China; ^2^ Forestry and Forest Products Research Institute 1 Matsunosato Tsukuba Ibaraki 3058687 Japan; ^3^ Organismal and Evolutionary Biology Viikki Plant Science Centre (ViPS) University of Helsinki PO Box 65 Helsinki 00014 Finland; ^4^ UNIROUEN INRAE ECODIV Normandie University Rouen 76000 France; ^5^ CAS Key Laboratory of Tropical Plant Resources and Sustainable Use Xishuangbanna Tropical Botanical Garden Chinese Academy of Sciences Menglun 666303 China; ^6^ Centre for Plant Ecology Core Botanical Gardens Chinese Academy of Sciences Xishuangbanna 666303 China; ^7^ Sugadaira Research Station Mountain Science Centre University of Tsukuba Sugadaira Kogen Ueda, Nagano 1278294 Japan

**Keywords:** biogeochemical cycle, functional traits, mesic ecosystems, model simulation, ultraviolet radiation

## Abstract

- Litter decomposition determines carbon (C) backflow to the atmosphere and ecosystem nutrient cycling. Although sunlight provides the indispensable energy for terrestrial biogeochemical processes, the role of photodegradation in decomposition has been relatively neglected in productive mesic ecosystems.
- To quantify the effects of this variation, we conducted a factorial experiment in the understorey of a temperate deciduous forest and an adjacent gap, using spectral‐attenuation‐filter treatments.
- Exposure to the full spectrum of sunlight increased decay rates by nearly 120% and the effect of blue light contributed 75% of this increase. Scaled‐up to the whole forest ecosystem, this translates to 13% loss of leaf‐litter C through photodegradation over the year of our study for a scenario of 20% gap. Irrespective of the spectral composition, herbaceous and shrub litter lost mass faster than tree litter, with photodegradation contributing the most to surface litter decomposition in forest canopy gaps. Across species, the initial litter lignin and polyphenolic contents predicted photodegradation by blue light and ultraviolet B (UV‐B) radiation, respectively.
- We concluded that photodegradation, modulated by litter quality, is an important driver of decomposition, not just in arid areas, but also in mesic ecosystems such as temperate deciduous forests following gap opening.

## Introduction

Litter decomposition, a critical step for carbon (C) and nutrient turnover, determines C balance in terrestrial ecosystems partially offsetting C input from primary production (Schlesinger & Bernhardt, 2013). Typically, litter decomposition is thought of as a biological enzymatic process mainly controlled by microorganisms (Swift *et al*., 1979). Climate (principally temperature and precipitation) and initial litter quality (directly affecting soil organisms), have been modelled extensively to predict litter decay rates (Gaxiola & Armesto, 2015). Empirical models can only explain up to 70% of the variation in decay rates in global terrestrial ecosystems (Parton *et al*., 2007). This implies that the models are importantly incomplete: other abiotic drivers or fundamental mechanisms in nature also contribute to this process. Obtaining a more‐complete picture of the drivers of litter decay is key to predicting how terrestrial C and nutrient cycles respond to climate changes.

Sunlight provides energy for photosynthesis to underpin ecosystem functioning in most terrestrial ecosystems. However, solar radiation can also directly break down organic matter through photochemical mineralization of complex macromolecules (e.g. lignin) that are able to absorb ultraviolet (UV) radiation (280–400 nm) and short‐wavelength visible light (Austin & Ballare, 2010), producing smaller organic compounds. This can indirectly benefit microbial activity (photofaciliation) by producing labile organic matter from recalcitrant compounds (Berenstecher *et al*., 2020). Photodegradation, the result of photochemical mineralization and photofaciliation driven by sunlight (Lin *et al*., 2018), is an important driver partially controlling the decay of litter in deserts, semi‐arid shrublands, grasslands, and savannas (Austin & Vivanco, 2006; Brandt *et al*., 2009; King *et al*., 2012; Day & Bliss, 2019). This potentially explains why biogeochemical models derived from mesic ecosystems always underestimate decay rates in drylands (Austin & Vivanco, 2006; King *et al*., 2012; Austin *et al*., 2016; Chen *et al*., 2016; Adair *et al*., 2017; Asao *et al*., 2018).

The relative magnitude of the photodegradation effect on decay rates partly depends on the spectral regions to which litter is exposed. In the past decades, UV radiation, and particularly UV‐B (280–315 nm), has been widely considered as the major driver of photodegradation in arid and semi‐arid ecosystems (Newsham *et al*., 1997; Gallo *et al*., 2009). Recently, this finding has been extended to peatlands (Foereid *et al*., 2018), Arctic, and alpine environments (Foereid *et al*., 2011). Although UV‐B radiation is estimated to increase litter mass loss by nearly 23% globally (King *et al*., 2012), it can also reduce decay rates through the inhibition of microbial decomposers activity (photoinhibition) (Pancotto *et al*., 2003). Moreover, although the initial lignin content is considered to be the main target of photochemical mineralization in litter, there is no evidence for a global relationship between lignin and UV photodegradation (King *et al*., 2012). This suggests that other spectral regions potentially play a key role in photodegradation. Indeed, relatively recent evidence revealed that blue and green light (400–550 nm) can alleviate the lignin bottleneck for litter decomposition more efficiently than UV‐B radiation (Brandt *et al*., 2009; Austin & Ballare, 2010; Austin *et al*., 2016). This implies that previous studies, focused solely on the UV region in drylands, may have underestimated the extent to which solar radiation affects decomposition (Austin *et al*., 2016). Most importantly, this critical finding suggests that photodegradation has the potential to drive C dynamics and nutrient cycling not only in drylands but also in mesic biomes at higher latitudes which receive less UV radiation. Nevertheless, the significance of photodegradation in mesic ecosystems is much less understood, particularly in dynamic light environments such as forest understoreys.

Temperate forests play a key role in determining the strength of the global terrestrial C sink, which depends on sunlight to support forest primary productivity (Leuchner *et al*., 2011). However, photodegradation has not been included in biogeochemical models in mesic ecosystems which typically have high leaf area index and relatively less radiation in general reaching the organic matter at the forest floor, despite it being accounted for in drylands at the same latitudes (Gallo *et al*., 2009; Brandt *et al*., 2010). However, the solar radiation reaching the forest floor varies greatly, as forests undergo succession, displaying spatially and temporally dynamic canopy and soil processes. In deciduous forest understoreys, seasonal canopy phenology causes large changes in light quality and irradiance through the year that potentially produce complex effects on litter decompostion (Pieristè *et al*., 2019, 2020a,b). Canopy gap dynamics created by a variety of natural or human disturbances (Čada *et al*., 2016), produce heterogeneous light environments in a forest that partially drive species diversity (Wang *et al*., 2020). This leads to variation in leaf structural and biochemical functional traits (e.g. lignin, nitrogen (N), polyphenols, and leaf mass area (LMA)), which may determine subsequent litter decomposition (Cornwell *et al*., 2008). However, little is known about the role of the high irradiance in gaps on decomposition and also which spectral regions most drive decomposition processes (Ma *et al*., 2017; Pieristè *et al*., 2019, 2020a,b; Marinho *et al*., 2020). It is also unclear whether photodegradation rates differ among plant growth forms, and if so, which initial litter traits best predict such changes. These uncertainties hamper our capacity to estimate the relative importance of photodegradation in litter decomposition, and eventually in C dynamics and nutrient cycling of forest ecosystems, consequently limiting our ability to forecast how climate and land‐use changes will affect these ecosystem processes.

We carried out a factorial experiment covering an area of *c*. 500 m^−2^ to study the relative effects of solar radiation on litter mass‐loss in the understorey of a temperate deciduous forest and an adjacent gap created by a clear‐cut. The relative contribution to decomposition of those spectral regions involved in photodegradation was quantified through spectral‐attenuation‐filter treatments created by custom‐made litterboxes (a total of 2304 replicate litterboxes). We identified litter traits that determined mass loss and predicted photodegradation by measuring the 12 typical structural and biochemical traits of initial litter. Our hypothesis was that photodegradation accelerates litter decomposition in this temperate forest ecosystem. Specifically, (1) the role of photodegradation would be greater in the gap than in the understorey, where cumulative irradiance was far lower due to canopy closure in summer; (2) those regions of shortwave visible light that are strongly absorbed by litter macromolecules and contribute most to spectral irradiance (i.e. blue and green light) would mainly drive photodegradation; (3) initial litter traits, e.g. lignin and phenolic contents, would predict differences in photodegradation between species due to their high energetic absorbance of shortwave solar radiation. Based on the experimental results we obtained, we simulated the potential effects of the photodegradation of leaf litter at the stand level in the studied forest.

## Materials and Methods

### Study site and litter collection

**Fig. 1:**
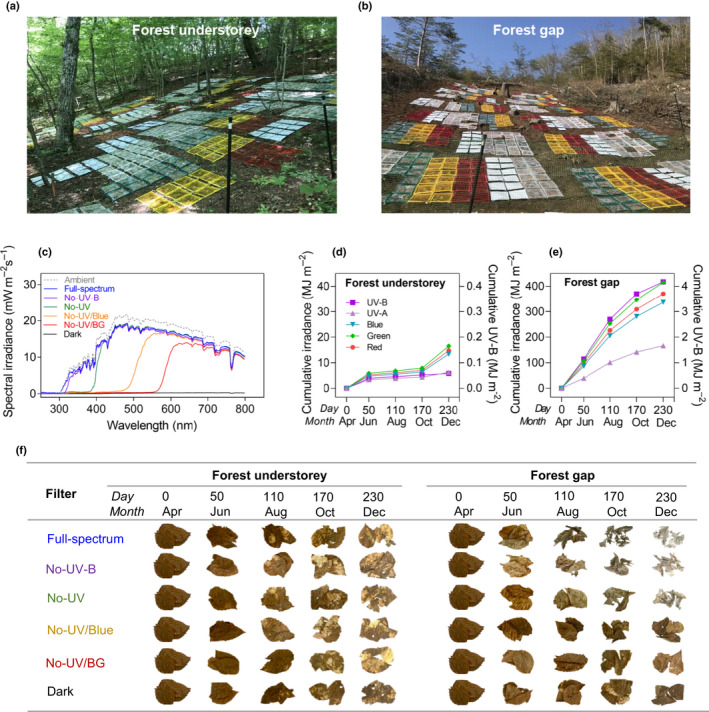
Field experiment is designed to test the importance of photodegradation for leaf‐litter decomposition in a temperate deciduous forest. Photographs of the experiment in the (a) understorey and (b) gap (7 June 2018). (c) Comparison of clear‐sky spectral photon irradiance at solar noon on 3 June 2018 in the forest gap under the attenuating filters in custom‐made litterboxes of different radiation treatments: (transmitting (1) > 280 nm (Full‐spectrum); (2) > 315 nm (No‐UV‐B); (3) > 400 nm (No‐UV); (4) > 500 nm (No‐UV/Blue); (5) > 580 nm (No‐UV/BG); (6) no light (Dark)). Cumulative irradiance in the (d) understorey and (e) gap, over 8 months of the decomposition experiment covering seasonal canopy dynamics (April—December 2018). (f) Photographs of cleaned, air‐dried leaf litter of one studied species (*Fagus crenata* Blume) from each filter treatment over the 8 months. Photographs taken at the same distance, and the background removed using Adobe Photoshop CS5.

The study site located in and around the Ogawa Forest Reserve (OFR, *c*. 100 ha) in the southern part of the Abukuma Mountain range in central Japan (36°56′N, 140°35′E, elevation 610 to 660 m above sea level (asl)). The mean annual temperature is 12.4°C, and annual precipitation is 1750 mm. The snow cover is 0–20 cm deep from January to March. The reserve is an old‐growth (at least 100–200‐year ‐old), cool‐temperate deciduous forest, dominated by *Quercus* spp., *Fagus* spp., *Acer* spp. To investigate effects of solar spectral radiation on litter decomposition, we established a 50 m × 50 m plot in the OFR understorey where the light environment was naturally impacted by seasonal canopy phenology, and a similar‐size plot in an unshaded gap created just outside the OFR (Fig. 1a,b) *c. *5‐km away. The gap plot was clear‐cut forest of similar composition to the understorey plot less than 1 year before the experiment in 2018. It offers a homogenous light environment and its proximity ensured a similar soil type and climate to the forest understorey plot. Both plots had a similar slope, aspect (south, 180°) and gradient 20°, and were protected by fences to exclude wild mammals (mainly boars).

We collected freshly senescent leaves from 12 species, with a range of trait values for variables including LMA and N content, from three growth forms (trees, shrubs, herbaceous; species list in Supporting Information Table S1) on 20–31 October 2017, in Sugadaira Research Station, Mountain Science Centre, University of Tsukuba, Sugadaira, Nagano, Japan (36°31′N; 138°20′E; 1340 m asl). Sampling a range of common species representing different growth forms allowed us to identify differentiation in leaf‐litter decay rates among growth forms in response to solar radiation. All litter samples were air‐dried in a darkened room (25°C, dehumidification mode of air conditioning) for 1 month. Each litter species, without petioles, was placed flat inside newspapers and pressed in a wooden frame from 25 December 2017 to 20 January 2018, ensuring homogeneous exposure to sunlight to avoid confounding factors. The pressed litter samples were kept in the dark at 25°C until their transfer to litterboxes.

### Experimental design and measurement

Litter materials were placed in an updated custom‐made litterbox based on our previous design (Pieristè *et al*., 2019). The litterbox frame and central pillar were made of sterile plastic straws (21 cm long, 18 cm wide and 1.3 cm diameter; Bihin, Japan) which made a space in the litterbox to avoid litter touching the filters during decomposition. Considering the effects of mesh size on mass loss from our previous study (Pieristè *et al*., 2019), the litterbox bases were made of sterile 2 mm mesh of polyethylene fibre, to avoid macrofaunal incursion while still allowing mesofauna access to the litter. Litterbox tops consisted of different filters: (1) Full‐spectrum treatment, with a fully transparent polythene film (0.05 mm thick, 3904CF; Okura, Marugame, Japan), transmitting approximately 95% of the whole solar spectrum > 280 nm; (2) No‐UV‐B treatment, attenuating UV‐B radiation < 315 nm (0.125‐mm‐thick polyester film, Autostat CT5; Thermoplast, Helsinki, Finland); (3) No‐UV treatment, attenuating all UV radiation < 400 nm (0.2‐mm‐thick Rosco E‐Color 226 filter, Westlighting, Helsinki, Finland); (4) No‐UV/Blue treatment, attenuating all UV and blue wavelengths < 500 nm (0.20‐mm‐thick Roscolux Supergel 312 filter); (5) No‐UV/Blue–Green (BG) treatment, attenuating all UV radiation and BG wavelengths < 580 nm (0.2‐mm‐thick Rosco E‐Color 135 Deep Golden Amber filter), (6) Dark treatment, attenuating all photosynthetically active radiation (PAR) and UV radiation (0.02‐mm‐thick polyethene film, white upper side and black lower side; Iwatani Materials Corp., Tokyo, Japan). Holes of 2‐mm diameter were drilled into the filter on a 1‐cm grid in order to allow moisture, air, and microbes to interact with the litter within. The solar spectrum was measured in each litterbox under a clear sky at solar noon on 3 June 2018 using a spectroradiometer (USR‐45DA; USHIO, Tokyo, Japan) (Fig. 1c; Table S2). We placed a single‐layer of nonoverlapping litter in the litterbox to make a homogenous total surface area of litter (*c.* 60% of the litterbox area), recorded by scanning each litterbox (Epson scanner, GT‐S600; Seiko, Nagoya, Japan). Leaf litter was placed with the adaxial epidermis facing up, and fixed flat in position on the bottom mesh using stainless‐steel staples (2–8 leaves per litterbox, weighing 0.2–1.0 g depending on the species). The whole experiment included 2304 litterboxes in total (12 litter species × 6 filter treatments × 2 plots × 4 collection times × 4 replicates) (Fig. 1c).

Litterboxes for 12 species within each treatment were randomly pinned to the soil surface in four blocks within each plot (understorey and gap) on 18 April 2018 before canopy flushing. Plots were checked every 2 weeks. Any debris on the litterboxes and nearby newly‐grown plants were removed in order to keep litterboxes unobstructed. Ambient UV‐B, UV‐A radiation, and PAR were continuously measured, integrated over 15‐min intervals using two calibrated broadband UV‐cosine sensors (UV‐B and UV‐A) (sglux GmbH, D‐12489, Berlin, Germany) and a quantum sensor (LI‐190SA; Li‐Cor, Lincoln, NE, USA) respectively, recorded with a data‐logger (LI‐1400; Li‐Cor). The spectral irradiance in litterboxes was checked during the experiment to ensure that all filters retained their specified spectral attenuations, using a Maya 2000 Pro array spectrometer (Ocean Optics Inc., Orlando, FL, USA), recently calibrated for maximum sensitivity in the solar UV and PAR regions of the spectrum (Hartikainen *et al*., 2018). Litter temperature was measured at 30‐min intervals using HOBO H8 Pro temperature loggers (Onset Computer Corp., Bourne, MA, USA). Soil samples (to 5 cm depth) under extra litterboxes were collected at solar noon (*n* = 12) once a month, and their gravimetric soil moisture content was calculated after oven drying (105°C, 72 h).

Four replicate litterboxes from each treatment combination were randomly collected on 7 June, 6 August, 5 October and 4 December 2018, after 50, 110, 170 and 230 d of the field experiment. These harvests were respectively timed to correspond with: canopy flush, completely closed canopy, leaf senescence, and autumn canopy opening (see litter photographs in Fig. 1f). Four of 12 species decomposed much faster than the rest, so were entirely collected over the first three dates: these species were two herbs, *Filipendula camtschatica* and *Pertya trilobata* and two shrubs, *Lindera obtusiloba* Blume and *Schisandra chinensis* (Fig. S4, see later; Table S1). After their retrieval, litterboxes were gently cleaned and air‐dried at the room temperature (dark, 25°C) for at least 1 month. The ash‐free (organic) mass and ash (inorganic) content were determined using a muffle oven (550°C for 5 h).

### Litter traits

For each species, prior to the start of the experiment we made initial trait measurements of the leaf litter assigned to five replicate litterboxes chosen at random (Table S1). LMA was determined based on litter area and oven‐dried weight of scanned leaves, using Fiji software (www.fiji.sc, imagej). Litter toughness was measured at the middle point of the lamina, avoiding the main veins, using a digital force gauge (DS2‐50 N; Imada, Toyohashi, Japan) together with a flat‐ended cylindrical steel punch (2 mm in diameter). The ground sample was also ashed at 550°C for 5 h. Nitrogen concentration [N] and carbon concentration [C] were determined using an elemental analyser (Vario MAX cube, Hanau, Germany). The Folin–Ciocalteu method was used to determine the concentration of total phenolics using tannic acid as a standard curve (Waterman & Mole, 1994). A proanthocyanidin assay was used to determine the content of condensed tannins with a standard curve prepared using cyanidin chloride, a commercially available anthocyanidin (Julkunen‐Tiitto, 1985). An improved acetyl‐bromide procedure was used to determine the lignin content, and the concentration of lignin was calculated from the fitted calibration curve (Fukushima & Hatfield, 2001). Sugar and starch content were determined using the anthrone method (Seifter *et al*., 1950).

### Ecosystem‐level litter production

Plant litter materials were collected monthly (from April to December) using 25 0.5 m^2^‐nylon‐mesh litter‐traps evenly distributed 20 m apart in a 1.2 ha‐plot (120 m × 100 m) of a 6‐ha permanent plot in the OFR from 2013 to 2017. The collected plant material was then divided into leaf, branch, seed, reproductive parts, and others, and their dry weight recorded. The annual leaf litter production of OFR was calculated using these litter trap data (SIN02.zip, downloaded from http://www.biodic.go.jp/moni1000/findings/data/index.html, Japanese Ministry of the Environment Monitoring Sites 1000 Project).

### Statistical analyses

Linear mixed‐effects model (LMM) was used to test the effect of three fixed factors (time, canopy openness, and filter treatment) and their interactions on mass loss, with species as the random factor. Block was dropped in the analysis, because it did not have significant effect on mass loss, but made the model singularity. The effects of specific spectral regions were analysed using pairwise contrasts (function glht, R package multcomp). One‐way ANOVA results of effects of filter treatments on mass loss at each collection time are shown for each comparison. The contrasts between the treatments transmitting solar radiation: > 280 nm vs > 315 nm, > 315 nm vs > 400 nm, > 400 nm vs > 500 nm, > 500 nm vs > 580 nm, > 580 nm vs dark, give the effect of UV‐B, UV‐A, blue, green, and red light, respectively; while the contrast > 280 nm vs dark, gives the effect of the full spectrum from UV‐B to red light. Benjamini–Hochberg's (BH) method (Benjamini & Hochberg, 1995) was used to correct these *P* values for multiple comparisons.

Litter mass loss was assumed to follow the exponential equation *X*_t_ = *X*_0_ *e^−kt^*, where *X*_0_ and *X*_t_ are the ash‐free dry mass at the beginning and at time *t*, *k* is the decay constant, calculated for each species based on the mean mass loss at each collection time. The spectral response ratios of mass loss, *RR*(mass loss), and of *k* values *RR*(*k*), between spectral contrasts, were calculated to examine the relative effect of photodegradation. As the *RR* value (e.g. *RR*_blue_) increases above 1.0, more mass is lost (i.e. the decomposition rate is faster), as attributable to a specific spectral region, e.g. blue light, compared with litter that did not receive blue light. LMM were used to examine the effects of three fixed factors, canopy openness, growth form, and filter treatment (or spectral region) and their interactions on *k* (or *RR*(*k*)), with species as the random factor. Differences between plots and among treatments were tested by Tukey's multiple comparison. Linear least squares method was used to analyse the correlation between initial traits and *k*, and that between initial traits and *RR*(*k*). The Yeo–Johnson transformation was applied where appropriate to ensure the normality of response variables (Yeo & Johnson, 2000). Photodegradation efficiency on mass loss or decay rate was calculated as *RR*(mass loss) or *RR*(*k*) divided by watt of energy irradiance received by litter.

The decay constants (*k*) for litter C were calculated for the studied tree species (*N* = 4) following the same equation earlier for litter mass loss *k* and then averaged for each treatment for each plot (forest understorey and gap). The annual litter C production was calculated multiplying the annual litter production for each year (2013–2017) in OFR by the mean initial litter C contents of the four tree species. The amount of leaf litter C remaining on the forest floor after 1 year of decay was first estimated by using the mean *k* for C under dark condition in the forest understorey, which indicates the C remaining after litter decomposition without photodegradation (mainly due to microbial activities). The amount of leaf litter C remaining were then modelled with the exponential equation *X*_t_ = *X*_0_ *e* ^−((1‐^ *^x^* ^)^ *^ki^* ^+^ *^xkj^* ^)1^. The annual leaf litter C production for each year (2013–2017) was plugged in for *X*_0_. The values of *ki* and *kj* are the mean *k* for C of under the full spectrum in the forest understorey and forest gap, respectively, to estimate the effect of photodegradation. The *X* takes 0 to 1, with 0.1 interval, assuming at fully closed canopy (forest understorey = 0.0) compared to a fully open canopy (forest gap = 1.0). Annual leaf litter C production was assumed to be constant even under the open canopy. This model estimated how much the decay of leaf litter C produced annually was affected by photodegradation depending on the proportion of open canopy created, based on scenarios of forest harvesting or disturbance intensity. Variation in C remaining among model results was tested using a one‐way ANOVA, and the relative difference between scenarios representing different proportions of gaps and continuous canopy cover with and without inclusion of photodegradation in the model was estimated using Tukey's multiple comparisons test. All statistical analyses were done in R v.3.6.3 (R Core Team (2020), Vienna Austria).

## Results

### Environmental characteristics of the study sites

Spectral irradiance in the forest understorey was dramatically modified though the year by the changing phenology of the forest canopy from leaf flushing to canopy closure and to leaf‐fall. However, in the gap created in 2018, 1 year before the experiment, sunlight followed the seasonal pattern typical of the mid‐latitudes in the Northern Hemisphere. On nearly 60% of days over the experimental period, sunlight in the gap reached a threshold irradiance of PAR > 1000 µmol m^−2^ s^−1^ (Figs 1e, S1). Whereas in the understorey, the closed canopy transmitted about 3.9%, 3.7% and 1.4% of above‐canopy PAR, UV‐A and UV‐B radiation, respectively, over the whole study period (Figs 1d, S1). Average litter temperature over the experiment differed between the gap (22.4°C) and the understorey (16.7°C) plots (Fig. S2). Soil water content measured gravimetrically was similar among filter treatments and between plots (Fig. S3).

### Litter mass loss

**Fig. 2:**
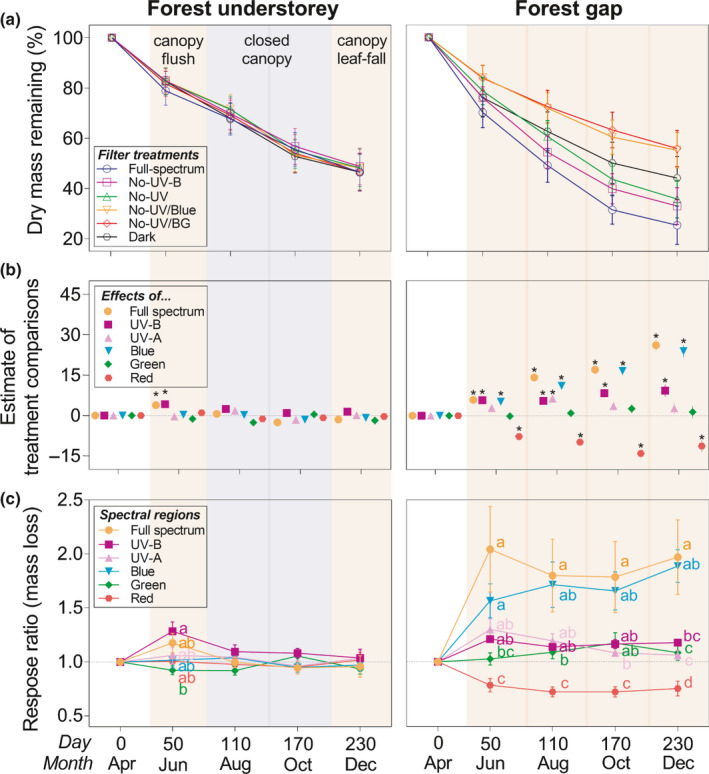
Solar spectral regions drive differences in mass loss from litter in the forest canopy gap and understorey. (a) Organic mass loss of leaf litter from 12 species (pooled) exposed to solar radiation in six filter treatments in the forest understorey and gap. (b) Pairwise comparisons for organic dry mass loss responding to filter treatments in 12 species’ leaf litter in the forest understorey and gap: *F*‐tests, with the BH method correction for multiple comparisons, were used to calculate the *P*‐values. Each point represents effects of spectral regions by comparing contrasts between treatments (see details in the Materials and Methods section). The asterisk indicates the significant difference between treatments. Details are shown in Supporting Information Table S4. (c) Response ratio of mass loss, *RR*(mass loss), to spectral regions (full spectrum, UV‐B, UV‐A, blue, green, red), calculated as the ratio of mass loss between pairs of spectral treatments over 8‐month decomposition. Different lowercase letters denote significant difference among spectral regions within a collection (*P* < 0.05), tested by least significant difference (LSD) multiple comparisons with BH method adjustment. Values are means across species (± SEM, *n* = 12). Four fast‐decomposing species were totally collected at the third time‐point (October) (Fig. S4), and the data at the final collection, calculated according to their decay rates (*k*), was interpolated in the mass loss curve in (a).

Over our field decomposition period (230 d), canopy openness played an important role in determining the rate of mass loss (*P* < 0.001, Table S3). The average litter mass loss across all treatments at final harvest was 58% in the forest gap, compared with 53% in the understorey (Fig. 2a). When effects of filter treatment were included, the pattern of mass loss diverged among filter treatments irrespective of species (Fig. S4) and was modulated by the canopy openness (Fig. 2a; Table S3). In the gap, litter receiving the full spectrum lost mass fastest (Fig. 2b; Table S4). Among the single spectral regions, blue light (No‐UV vs No‐UV/Blue treatment) and UV‐B radiation (Full‐spectrum vs No‐UV‐B treatment) enhanced mass loss, and the former's effect on mass loss was almost equal to that of the full spectrum (Full‐spectrum vs Dark treatment). Differences among spectral regions became most evident towards the end of the experimental period, e.g. mass loss up to 75%, 67%, and 45% in the Full‐spectrum, No‐UV‐B, and No‐UV/Blue treatments, respectively (Fig. 2a), as illustrated in the litter photographs (Fig. 1f). In contrast, enhanced mass loss in the understorey was only observed in Full‐spectrum vs No‐UV‐B and Dark treatments after the first 50 d of decomposition prior to canopy closure, but not subsequently (Fig. 2b; Table S4), showing that even in early spring UV‐B radiation can affect decomposition (*z* = 4.87, *P* < 0.001; *z* = 5.26, *P* < 0.001, respectively; Table S4). Overall, these results reveal how dramatically photodegradation modified litter decomposition as well as the interactions between photodegradation, forest canopy openness and phenology.

We calculated the spectral response ratio of mass loss at each harvest, *RR*(mass loss), to quantify the relative effects of spectral regions on photodegradation (Table S5). The values of *RR*_full spectrum_ (1.2) and *RR*_UV‐B_ (1.3) in the understorey were both positive over the first 50 d, while significant variation was not detected among other spectral regions (*P* > 0.05) (Fig. 2c). However, the spectral regions had a large effect in the gap (Fig. 2c). Here, *RR*_full spectrum_ (2.0) and *RR*_blue_ (1.9) were consistently higher than other spectral treatments during the experiment (Fig. 2c). These results illustrate that the contribution of photodegradation to mass loss was subtle in the understorey, but pronounced in the gap, with a large effect of blue light compared with other spectral regions. Furthermore, considering photodegradation efficiency, i.e. mass loss per watt of energy irradiance litter received, the spectral response ratio in the understorey was greater than that in the gap (Fig. S5; Table S5). Photodegradation efficiency was much higher for UV‐B radiation than blue light, even in the forest gap where blue light drove photodegradation.

### Decay constant

**Fig. 3:**
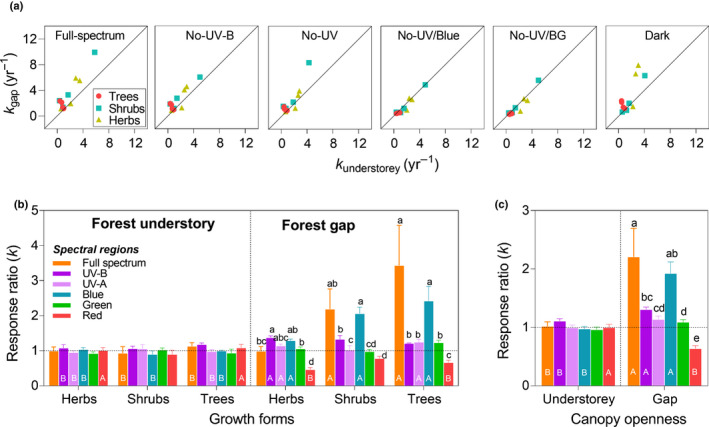
The importance of solar radiation for decomposition rate is modified by canopy openness and plant growth forms. (a) The 1 : 1 line of *k* values between the forest understorey and gap for each filter treatment. (b) Response ratio of *k* values, *RR*(*k*), to spectral regions, calculated as the ratio of *k* values between pairs of spectral treatments at the functional‐group level (mean ± SE, *n* = 4). (c) *RR*(*k*) at the plot level (mean ± SE, *n* = 12). Different lowercase and uppercase letters denote significant difference among spectral regions within a growth form and plot (*P* < 0.05), and between plots in response to the same spectral regions, respectively.

We integrated the rates of litter mass loss over time, expressing decomposition as a decay constant (*k*). Overall, *k* tended to be higher for herb and shrub litter than for tree litter (Fig. S6a,b), but the response varied among species to such an extent that filter treatments had an insignificant interaction with growth forms (*F* = 1.7; *P* = 0.085; Table S6). There was a significant interaction between canopy openness and filter treatment (*F* = 12.5, *P* < 0.001; Table S6), but *k* did not significantly differ among filter treatments in the forest understorey. However, in the gap, *k* decreased from Full‐spectrum (3.21 yr^−1^) to No‐UV (2.26 yr^−1^) and significantly to No‐UV/BG (1.36 yr^−1^) treatments (Fig. S6c). In the understorey, *k* values were markedly lower than in the gap, except for No‐UV/Blue and No‐UV/BG treatments (Figs 3a, S6c). In addition, *k* in the Dark treatment in the gap was higher than in the understorey Dark treatment (Fig. S6). Evidence for herbivorous insects was found which are likely to have affected this understorey Dark treatment sample (see Fig. S7).

The relative photodegradation effect on decay rate, *RR*(*k*), varied significantly depending on canopy openness, growth form, spectral region, and their interactions (Table S7). The value of *RR*(*k*) was similarly close to 1.0 among spectral regions in the understorey, and only the response ratio for UV‐B exposure (*RR*_UV‐B_) was slightly higher (on average 1.1; Fig. 3c). In the gap, *RR*(*k*) had distinct functional‐group dependency. The values of *RR*_full spectrum_ and *RR*_blue_ were higher than other spectral regions for shrub litter (on average 2.2 and 2.1) and tree litter (on average 3.4 and 2.4) but not for herb litter (1.0 and 1.2 on average: Fig. 3b). In contrast, *RR*_red_ was negative and tended to be lowest for all three growth forms (Fig. 3b). Overall, full spectrum photodegradation in the gap greatly accelerated the decay rate to 120% compared with dark conditions: this effect was mainly due to blue light (*c. *75%); however, red light slowed the decay rate by 40% (Fig. 3c). The response efficiency per watt of energy irradiance received by litter in the understorey was much greater than in the gap (Fig. S8; Table S7); it was also much higher for UV‐B radiation than blue light, irrespective of canopy condition.

### Relationship between initial litter traits and photodegradation

**Fig. 4:**
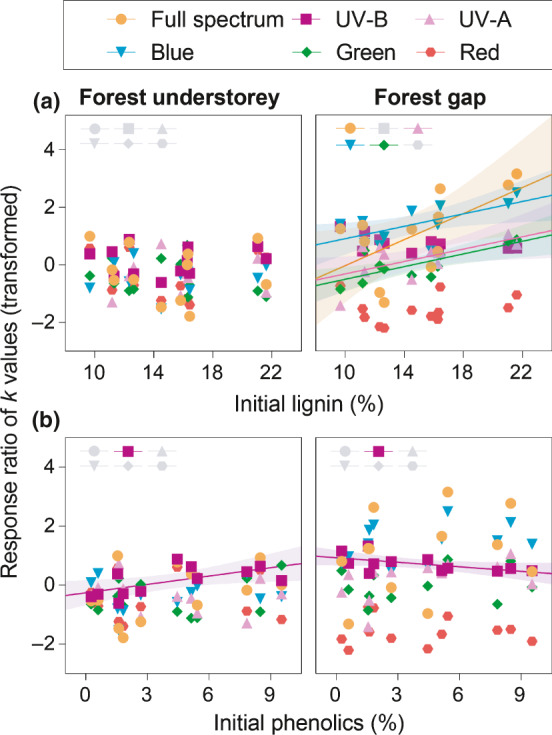
Do initial lignin and total phenolics content of litter predict photodegradation. (a) Correlation between initial lignin content and response ratio of *k* values for spectral contrasts in the forest gap. (b) Positive and negative correlations between initial total phenolic content and the response ratio of *k* values responding to UV‐B in the forest understorey and gap, respectively. Values are means (*n* = 5) of each litter type for initial traits. The *k* values were transformed by the Yeo–Johnson power transformation. Solid coloured lines denote significant relationships (*P* < 0.05). The coloured shading is used to show the 95% confidence bands of the best‐fit line, if the correlation is significant (± SE). Detailed coefficient and *P* values are in Supporting Information Table S8.

We further analysed relationships between initial traits and *RR*(*k*) to quantify which traits make litter more responsive to photodegradation from UV radiation and shortwave visible light, based on relationship between initial litter traits and the decay constant (Notes S1). We found that initial lignin content was the strongest predictor of photodegradation in the gap, but not in the understorey. This relationship held in the forest gap for all spectral regions studied except for UV‐B and red light (*R* = 0.60 ~ 0.74, *P* < 0.05; Fig. 4a; Table S8), suggesting that high‐lignin litter was most prone to photodegradation from UV‐A, blue, and green light. This was not the case for the forest understorey, where *RR*(*k*) was positively correlated with the total phenolic content in response to UV‐B (*RR*_UV‐B_) (*R* = 0.63, *P* = 0.029; Table S8). This relationship shifted from positive to negative in the gap (*R* = −0.58, *P* = 0.050; Fig. 4b; Table S8). A similar pattern was visible in tannin content, where the correlation was significant in the gap (*R* = −0.62, *P* = 0.031) but not the understorey (*R* = 0.52, *P* = 0.083; Table S8). This illustrated that leaf litter with higher polyphenolic content promoted UV‐B photodegradation in the understorey but suppressed it in the gap.

### Photodegradation effects on forest leaf litter stocks

**Fig. 5:**
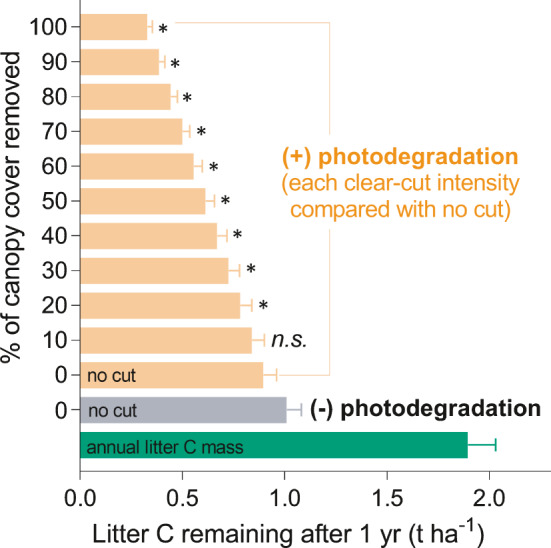
Photodegradation increases in the amount of carbon (C) released from decomposing leaf litter at the ecosystem level when gap creation is considered. Annual litter C mass (green bar) was calculated from litter produced over 5 years (2013–2017). The amount of leaf litter C lost after 1‐yr of decay was modelled with (yellow bars) or without (grey bar) the photodegradation effect, under scenarios of gap. Values are means across years (mean ± SD, *n* = 5). Asterisks represents the significant difference (*P* < 0.05) in the litter C loss at different clear‐cut intensities from 10% to 100% of forest cover compared with continuous forest cover (‘no cut’ or 0% cut) with photodegradation; n.s., no significance (*P *> 0.05), estimated by Tukey's multiple comparisons test.

To test the potential effect of photodegradation on forest leaf litter stocks at the stand level, we calculated annual leaf litter production (4.0 t ha^−1^ yr^−1^) and C production (1.9 t ha^−1^ yr^−1^) for a forested area of 1.2 ha of the OFR for the last 5 years (2013–2017). We estimated the extent to which photodegradation affected the decay of leaf litter after 1 yr under various scenarios that simulated gap creation due to logging, based on the decay rates of tree litter (Fig. S6b) and the assumption that the annual leaf litter production is constant even under scenarios of cut‐clear gap creation. Photodegradation in gaps significantly modified litter decay at the forest stand level (*F* = 20.76, *P* < 0.001, Fig. 5). After 1 yr, the amount of leaf litter C remaining under continuous forest cover (no gaps) was similar, 0.9 and 1.0 t ha^−1^ in the dark vs exposed to the full spectrum of sunlight, respectively. This suggests that, in the understorey under continuous forest canopy cover photodegradation does not significantly change the annual C input from fresh leaf litter at the ecosystem level. However, scenarios of cut‐clear gap creation reducing canopy cover by 20%, 50% and 100% caused a significant increase in C loss from leaf litter of 13%, 32% and 63%, respectively, indicated by the amount of litter C remaining (0.8, 0.6, and 0.3 t ha^−1^) after 1‐yr decay compared to continuous cover (1.0 t ha^−1^) (Fig. 5).

## Discussion

Our study found that photodegradation plays a key role in driving surface litter decomposition in a temperate forest ecosystem. The relative importance of each spectral region is strictly dependent on canopy openness, which also directly determines the amount of radiation reaching the litter surface (Figs 1d,e, 2a); the difference among spectral treatments becomes larger towards the end of the summer in the gap (Fig. 2b,c). In the forest gap, exposure to the full spectrum of sunlight stimulated litter decay rates nearly 120% across litter from a wide diversity of species (Fig. 3c). This result implies that photodegradation has the potential to increase leaf litter turnover on the forest floor following greater gap opening, when the decrease in forest cover exposes litter to higher solar irradiance (Fig. 5). This result is consistent with findings from arid and semi‐arid ecosystems that identify photodegradation as the main driver of C turnover (Austin & Vivanco, 2006; King *et al*., 2012; Austin *et al*., 2016; Liu *et al*., 2018; Day & Bliss, 2019; Berenstecher *et al*., 2020). However, the magnitude of the effects in our study tended to be greater than findings from those environments: for instance the 60% increase in decomposition due to sunlight in semi‐arid Patagonian steppe (Austin & Vivanco, 2006), or the 23% increase across biomes (King *et al*., 2012). This may result from a difference in litter quality between biomes; leaf litter across species used in our study was generally thinner (LMA, 54 g m^−2^) but had a higher lignin content (15%) than that from drylands (200 g m^−2^ and 10%, respectively) (King *et al*., 2012). Critically, our study highlights the importance of blue light, accounting for 75% of the total photodegradation (Fig. 3c), over that of UV‐B radiation, which has often been considered as the main spectral region responsible for this process. This finding is consistent with a recent study identifying the effect of visible (BG) light on litter decomposition in a semi‐arid ecosystem (Austin *et al*., 2016), although that study does not separate the contributions of each spectral region. These findings suggest that out of all the spectral regions, the irradiance of blue light may best determine the sensitivity of litter decay to photodegradation across terrestrial ecosystems.

In the understorey, the time‐course of litter decay patterns was very similar among all filter treatments, particularly after spring‐time canopy flush that dramatically reduces the received irradiance (Figs 1d, 2a). Exposure to the full spectrum before canopy closure in the spring still promoted mass loss in the understorey (Fig. 2b), and comparing the effects of spectral regions revealed the importance of UV‐B radiation in this response. This underlines that photodegradation mainly occurs when the canopy is open in the understorey. Nevertheless, despite the smaller contribution of photodegradation and cooler temperatures, the absolute mass loss across all treatments in the understorey was not much slower than that in the gap (Fig. 2a). A similar result has been found in other mesic ecosystems, e.g. tropical and subtropical forests (Ma *et al*., 2017; Marinho *et al*., 2020), and may imply that increased humidity where temperature and irradiance are lower offsets these drivers of decomposition. Classical models of C turnover may function well for litter in understorey shade, where soil moisture is consistently high and temperature stable (Figs 3, S2) favouring high rates of microbial decomposition.

The succession of decomposer microbes during the decomposition process is intuitively visible when from images of surface litter (Fig. 1f). During the early phase of litter decomposition, bacteria are facilitated by the presence of micromolecules resulting from direct photomineralization, before canopy closure in the understorey (Pieristè *et al*., 2020); while macromolecules, particularly recalcitrant lignin, remain to be decomposed at a later phase by fungi, e.g. visible as white areas colonized by white rot fungus (Osono & Takeda, 2001). In contrast, strong sunlight and drastically fluctuating temperatures and moisture in the gap may disrupt the microbial community and inhibit microbial decomposition (Pancotto *et al*., 2003), apparent in the distinct differences in litter images between the understorey and gap (Fig. 1f). In addition, although average litter temperature in the gap (22.4°C) was higher than under the canopy (16.7°C) (Fig. S2), this difference might make a minor contribution to difference in litter decay rates between sites of contrasting canopy openness. Low temperature by itself (< 25°C) leads to minor thermal degradation (van Asperen *et al*., 2015), resulting in relatively low photochemical emission even in conjunction with high solar irradiance (Day & Bliss, 2019); whereas microbial activity induced by relatively high temperature may be counterbalanced by photoinhibition in the gap as discussed earlier. The biotic effect found in the gap, where mass loss was high in the dark treatments, was probably caused by relatively high microbial decomposition or decomposer herbivory (Figs 3, S6, S7), because micro‐ and meso‐fauna prefer darker environments (Lin *et al*., 2018; Pieristè *et al*., 2019, 2020a,b). Hence, litter decomposition tends to be driven by microbial decomposition in the understorey and by photodegradation in the gaps of mesic ecosystems, with canopy openness being the key factor determining the balance between these two processes.

Changing litter input from different plant growth forms according to canopy openness, affecting the contribution from canopy and understorey species, can also modify the importance of photodegradation in decomposition. Herbaceous and shrub litter lost mass much faster than tree litter, irrespective of the filter treatment in the understorey (Fig. S6), attributable to their low toughness and low C and lignin content (Table S1), which are structural and persistent compounds affecting decomposability (Pieristè *et al*., 2020a,b). In contrast, tougher litter from trees with a high content of recalcitrant compounds is more prone to photodegradation once exposed to sunlight in the gap (Fig. 3). Our finding that blue light is particularly effective in the photodegradation of tree litter with low decomposability is consistent with results of a recent study comparing three tree species in a European beech forest understorey (Pieristè *et al*., 2019). This implies that trees, dominating aboveground biomass in forests, promote local C and nutrient cycles through litter decomposition and alter ecosystem function following gap creation.

Initial contents of lignin and polyphenolic compounds in litter were identified as the strongest predictors of the relative effect of photodegradation on decomposition. Our results confirm the dual role of lignin: it is subject to photochemical mineralization when exposed to direct sunlight (particularly blue light) (Figs 3, 4), because of its strong absorbance particularly in the blue region compared to the UV‐B region (Austin & Ballare, 2010); this contrasts with the well‐known role of lignin in slowing decay rates across forest ecosystems due to its recalcitrance to microbial enzymatic degradation (Swift *et al*., 1979). Specifically responding to UV‐B radiation, however, polyphenolic compounds (e.g. total phenolics) might also have a dual role affecting litter decomposition, as inferred from their opposing positive and negative correlations with UV‐B photodegradation in the understorey and gap, respectively (Fig. 4b). The accumulation of polyphenolic secondary metabolites in the leaf epidermis generally functions as a UV‐screen reducing its penetration into the leaf mesophyll (Searles *et al*., 2001). These optical properties of leaves may persist during the early phase of litter decomposition (Pieristè *et al*., 2020) and could provide protection from UV radiation to microbial decomposers in the understorey where the biotic effect is dominant (Pancotto *et al*., 2003), but would also reduce the UV‐B enhancement of moss loss in the gap where photochemical mineralization dominates; a result and mechanism that is consistent with findings from controlled conditions (Pieristè *et al*., 2020). Furthermore, solar spectral irradiance and composition can also directly shape the structural and biochemical traits of living leaves (Wang *et al*., 2020), which may potentially amplify these photodegradation effects. These findings improve our understanding of the role of photodegradation in C and nutrient dynamics in forest ecosystems.

Classical models focusing on climatic factors are generally accepted to be good predictors of litter decomposition in the temperate forest ecosystems (Gaxiola & Armesto, 2015). Solar radiation has not been included in these models until now (except for a few studies in the dryland systems, i.e. Chen *et al*., 2016; Adair *et al*., 2017; Asao *et al*., 2018), even though it directly breaks‐down organic matter and may affect decomposition processes in forests. Our results illustrate that when gaps open in the forest canopy, photodegradation overrides temperature and moisture as a driver of leaf litter decomposition. Scaling up to the ecosystem level, 1‐yr exposure to the full spectrum of sunlight through gap creation dramatically reduces the retention and recycling of C from leaf litter into the forest soil (Fig. 5). Meanwhile, it increases C flow back to the atmosphere by up to 63% relative to a continuous forest understorey, because of the increased incident of solar irradiance on surface litter. This suggests that accounting for photodegradation would significantly improve predictions of C loss from mesic forest ecosystems, consistent with its recent inclusion in the equivalent calculations for dry grassland ecosystems (Adair *et al*., 2017; Asao *et al*., 2018). Our estimate is based only on tree litter. Caution is needed in estimating the importance of photodegradation from this scenario, since the deeper layers of litter on the forest floor are not directly exposed to sunlight and gap creation also changes the amount of composition of litter input to the ecosystem. However, surface layer photodegradation may have potential priming effects and consequently promote litter decomposition even once it is covered by new litter, though this hypothesis has not been tested because very few photodegradation studies take into account litter layer thickness (Mao *et al*., 2018).

Global forests cover about 31% of the land area and their vegetation and soil contain nearly half of the terrestrial biosphere C stock (FAO, 2020), but as nonequilibrium ecosystems they are continuously subject to a variety of disturbances, e.g. wind, landslide or logging (Čada *et al*., 2016). Thus, the omission of photodegradation from classical models implies that a considerable proportion of their C loss may have not been identified (Austin & Vivanco, 2006), though identifying the relative importance of photochemical mineralization and photofacilitation requires further research. In this respect, the finding that blue light, rather than UV radiation, dominates photodegradation in mesic ecosystems as well as arid ecosystems is of high importance as it broadens our knowledge of the ubiquity over biomes of photodegradation as a contributing driver of decomposition. The relevant role of photodegradation highlighted by our study, may explain why C and nutrient cycling are underestimated by the empirical models in global terrestrial ecosystems (Parton *et al*., 2007). Interactions of stratospheric ozone, climate (e.g. cloud cover and air quality), and land use (e.g. deforestation) are projected to modify the exposure of terrestrial organic matter to solar radiation (EEAP, 2019). Therefore, a better understanding how sunlight controls C and nutrient dynamics in various ecosystems is essential to make accurate assessments of how biogeochemical cycles might respond to climate changes.

## Author contributions

QWW, HK and TMR conceived and designed the experiment. QWW and TK collected leaf litter. QWW measured the morphological and chemical traits together with CL, QWW and HK set up the field decomposition experiment. QWW managed and retrieved litterboxes, and determined ash‐free mass loss. QWW, TMR and MP measured spectrum of field light environments and litterboxes. HK did the modelling test. QWW did statistical analysis and wrote the draft of the manuscript, and the remaining co‐authors did the revision.

## Supporting information


**Fig. S1** Spectral irradiance in the temperate deciduous forest understorey vs the gap.
**Fig. S2** Temperature variation in litterboxes in the understorey vs the gap.
**Fig. S3** Soil moisture under litterboxes in the understorey vs the gap.
**Fig. S4** Organic mass loss of 12 l species over 8 months.
**Fig. S5** Mass loss per Watt of the energy irradiance litter receives.
**Fig. S6** Growth forms interacting with canopy openness modify litter decay rates.
**Fig. S7** Herbivory may explain the high litter decay rate in Dark treatment.
**Fig. S8** Shorter‐waveband spectral regions per Watt of the energy irradiance litter receives.
**Notes S1** Relationship between initial litter traits and the decay constant
**Table S1** Initial traits of freshly senescent leaves among 12 species.
**Table S2** Spectral quality of filters.
**Table S3** Linear mixed‐effects model (LMM) results on litter mass loss.
**Table S4** The effect of individual spectral regions on litter mass loss.
**Table S5** LMM results on the response ratio of mass loss.
**Table S6** LMM results on *k* values.
**Table S7** LMM results on response ratio of *k* values and its values per watt of energy irradiance litter received.
**Table S8** Correlations between initial litter traits and response ratio of *k* values in six spectral regions.
Please note: Wiley Blackwell are not responsible for the content or functionality of any Supporting Information supplied by the authors. Any queries (other than missing material) should be directed to the *New Phytologist* Central Office.

## Data Availability

All data needed to evaluate the conclusions in the article are present in the article and/or the Supporting Information. Additional data related to this article may be requested from the corresponding author (QWW).
