# Supplementary material for: The contribution of photodegradation to litter decomposition in a temperate forest gap and understorey
Source: New Phytol. 2020 Nov 30;229(5):2625–36. doi: 10.1111/nph.17022 (PMC7898645; doi:10.1111/nph.17022)
Supplement: Supplementary file 1 — Fig. S1 Spectral irradiance in the temperate deciduous forest understorey vs the gap. Fig. S2 Temperature variation in litterboxes in the understorey vs the gap. Fig. S3 Soil moisture under litterboxes in the understorey vs the gap. Fig. S4 Organic mass loss of 12 l species over 8 months. Fig. S5 Mass loss per Watt of the energy irradiance litter receives. Fig. S6 Growth forms interacting with canopy openness modify litter decay rates. Fig. S7 Herbivory may explain the high litter decay rate in Dark treatment. Fig. S8 Shorter‐waveband spectral regions per Watt of the energy irradiance litter receives. Notes S1 Relationship between initial litter traits and the decay constant Table S1 Initial traits of freshly senescent leaves among 12 species. Table S2 Spectral quality of filters. Table S3 Linear mixed‐effects model (LMM) results on litter mass loss. Table S4 The effect of individual spectral regions on litter mass loss. Table S5 LMM results on the response ratio of mass loss. Table S6 LMM results on k values. Table S7 LMM results on response ratio of k values and its values per watt of energy irradiance litter received. Table S8 Correlations between initial litter traits and response ratio of k values in six spectral regions. Please note: Wiley Blackwell are not responsible for the content or functionality of any Supporting Information supplied by the authors. Any queries (other than missing material) should be directed to the New Phytologist Central Office. [file NPH-229-2625-s001.pdf]

Article acceptance date: 14 October 2020

Supplementary information

The contribution of photodegradation to litter decomposition in a temperate forest gap and understorey

Qing-Wei Wang[1,2*], Marta Pieristè[3,4], Chenggang Liu[5,6], Tanaka Kenta[7], Thomas Matthew Robson[3*], Hiroko Kurokawa[2*]

Notes S1: Relationship between initial litter traits and the decay constant

Fig. A Initial litter traits per dry mass predict decay rates under the six filter treatments.
Fig. B Initial litter traits per litter area predict decay rates under the six filter treatments.

Table A Correlations between initial litter traits and *k* values in six filter treatments.

Fig. S1 Spectral irradiance in the temperate deciduous forest understorey vs. the gap.
Fig. S2 Temperature variation in litterboxes in the understorey vs. the gap.
Fig. S3 Soil moisture under litterboxes in the understorey vs. the gap.
Fig. S4 Organic mass loss of 12 litter species over eight months.
Fig. S5 Mass loss per Watt of the energy irradiance litter receives.
Fig. S6 Growth forms interacting with canopy openness modify litter decay rates.
Fig. S7 Herbivory may explain the high litter decay rate in Dark treatment.
Fig. S8 Shorter-waveband spectral regions per Watt of the energy irradiance litter receives.

Table S1 Initial traits of freshly senescent leaves among 12 species.
Table S2 Spectral quality of filters.
Table S3 Linear Mixed-Effects Models (LMM) results on litter mass loss.
Table S4 The effect of individual spectral regions on litter mass loss.
Table S5 LMM results on the response ratio of mass loss.
Table S6 LMM results on *k* values.
Table S7 LMM results on response ratio of *k* values and its values per Watt of energy irradiance litter received.
Table S8 Correlations between initial litter traits and response ratio of *k* values in six spectral regions.

Notes S1

Relationship between initial litter traits and the decay constant

To identify potential litter traits that could predict the photodegradation rate, we measured the initial litter traits for the species chosen for the experiment (Table **S1**). Among 12 common traits, toughness and [C] of initial litter were common predictors of decomposition rate, regardless of spectral filter treatment or canopy openness (R = -0.92 ~ -0.59, $P < 0.05$; Fig. **Aa**, **b**; Table **A**). Lignin content was also negatively correlated with k in all treatments except for the Full-spectrum and No-UV-B treatments in the gap (Fig. **Ac**; Table **A**). The k value was correlated with lignin:N ratio, but not with [N] and C:N ratio, in the Dark treatment in the understorey, and in the No-UV/Blue, No-UV/BG and Dark treatments in the gap (Fig. **Ad**; Table **A**). Ash content was the only trait that correlated positively with k values in the No-UV/Blue, No-UV/BG and Dark treatments in the understorey (Table **A**), while it correlated with in all treatments except for No-UV/BG and Dark treatments in the gap (Fig. **Ae**). The k values were not correlated with total phenolic content but were negatively correlated with tannin content in the understorey Dark treatment (Fig. **Af**). The k values were also negatively correlated with LMA, starch, and sugar contents in the gap depending on the filter treatment (Fig. **Ag, h, i**). Significant correlations above tended to be weak or disappear when litter chemical traits were expressed per litter area (Fig. **B**; Table **A**).

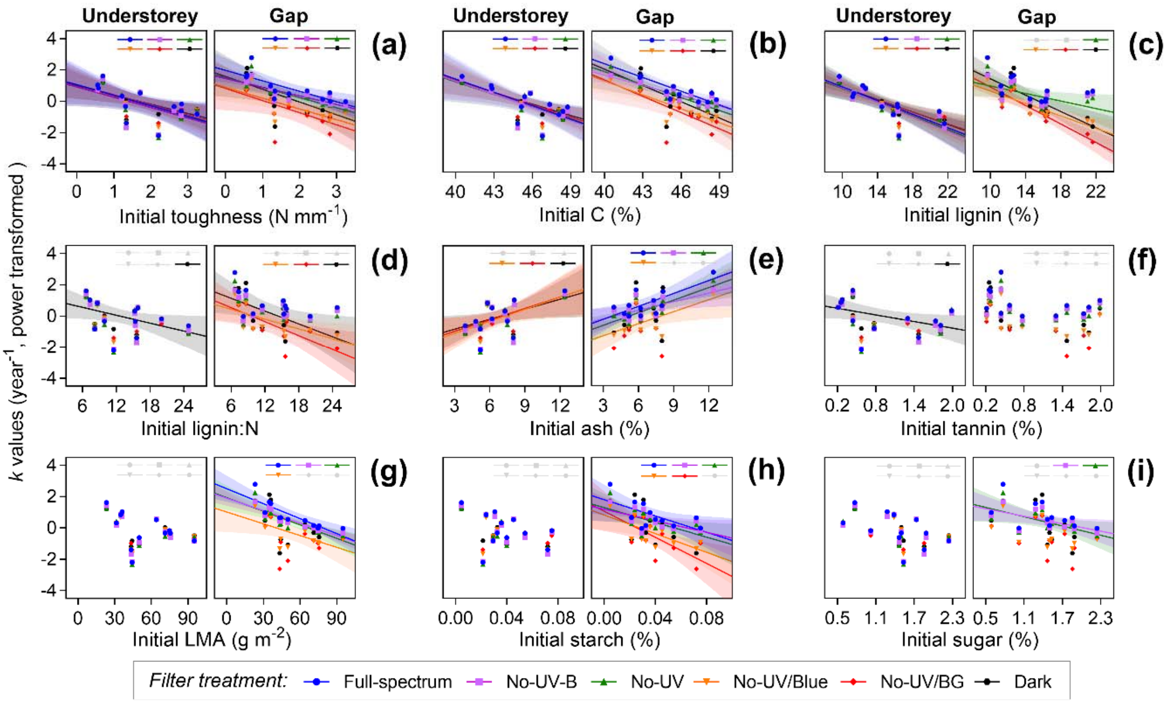

Fig. A Initial litter traits per dry mass predict decay rates under the six filter treatments differently depending on canopy openness. Relationship between initial traits (**a**) litter toughness, (**b**) carbon content, (**c**) lignin content, (**d**) lignin: N ratio, (**e**) ash content, (**f**) tannin content, (**g**) litter leaf mass area (LMA), (**h**) starch concentration, (**i**) sugar concentration and *k* values in the forest understorey and gap in the studied forest in central Japan (36°56'N, 140°35'E). Values are means (n = 5) of each litter species for initial traits. *k* values, sugar, and starch contents were normally transformed by the Yeo-Johnson power transformation. Solid color lines denote significant relationships ($P < 0.05$). The coloured shading is used to show the 95% confidence bands of the best-fit line, if the correlation is significant (+/- SE). Detailed coefficient and *P* values are in Supplementary Table **S8**.

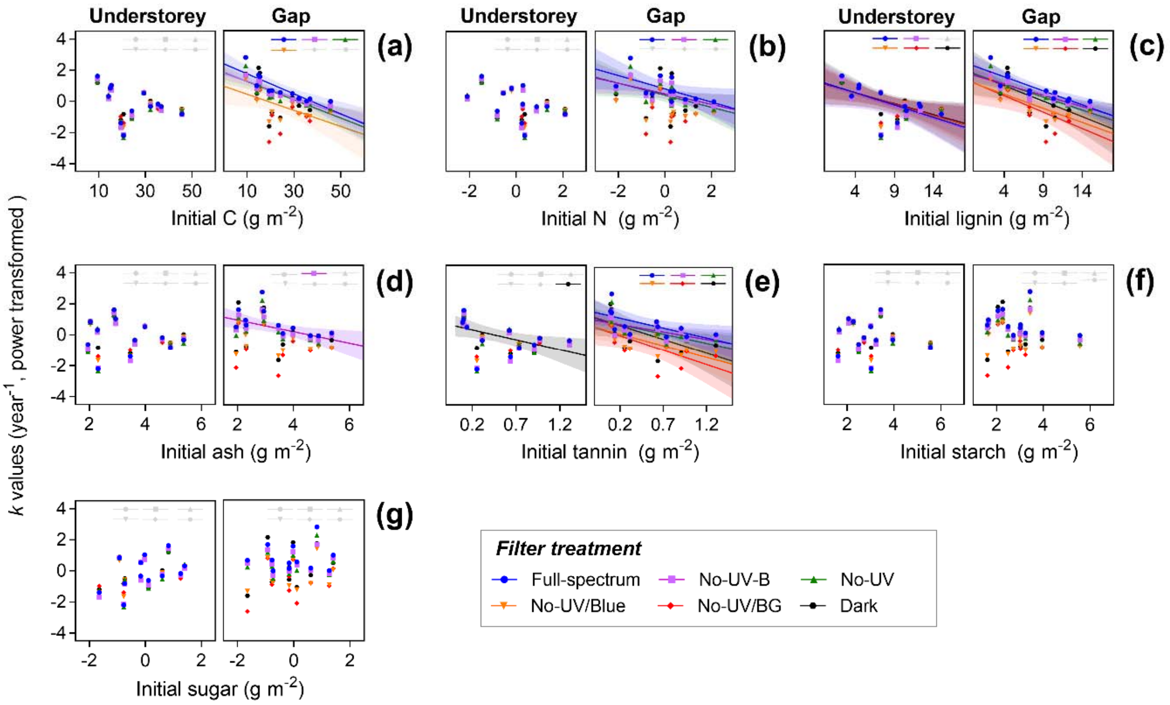

Fig. B Initial litter traits per litter area predict decay rates under the six filter treatments differently depending on canopy openness. Relationship between initial traits (**a**) carbon content, (**b**) nitrogen content, (**c**) lignin content, (**d**) lignin ash, (**e**) tannin content, (**f**) starch content, (**g**) sugar content and *k* values in the forest understorey and gap in the studied forest in central Japan (36°56'N, 140°35'E). Values are means (n = 5) of each litter species for initial traits. *k* values, nitrogen and sugar contents were normally transformed by the Yeo-Johnson power transformation. Solid color lines denote significant relationships ($P < 0.05$). The coloured shading is used to show the 95% confidence bands of the best-fit line, if the correlation is significant (+/- SE). Detailed coefficient and *P* values are in Supplementary Table **S8**.

Table A Correlations between initial litter traits (per unit dry mass and litter area) and *k* values (decay constant) in six filter treatments. Values are coefficients of determination (*R*) and *P*-values (n = 12). Normal and Italic values denote positive and negative correlations, respectively, while the bold shading denotes significant correlations (*P* <0.05).

Plots	Initial traits	Full-spectrum		No-UV-B		No-UV		No-UV/Blue		No UV/BG		Dark	
		R	*P*	*R*	*P*	*R*	*P*	*R*	*P*	*R*	*P*	*R*	*P*
Forest understorey													
	**Toughness (N mm^{-1})**	***-0.60***	***0.040***	***-0.60***	***0.040***	***-0.59***	***0.042***	***-0.63***	***0.028***	***-0.70***	***0.011***	***-0.70***	***0.011***
	LMA (%)	-0.38	0.224	-0.34	0.285	-0.32	0.306	-0.35	0.269	-0.45	0.144	-0.41	0.187
	**Ash (%)**	0.57	0.055	0.55	0.064	0.55	0.062	**0.62**	**0.033**	**0.63**	**0.028**	**0.59**	**0.041**
	**Lignin (%)**	***-0.76***	***0.004***	***-0.82***	***0.001***	***-0.80***	***0.002***	***-0.87***	***0.000***	***-0.83***	***0.001***	***-0.93***	***0.000***
	Phenolics (%)	-0.17	0.598	-0.26	0.419	-0.21	0.521	-0.30	0.345	-0.36	0.255	-0.45	0.142
	**Tannin (%)**	-0.33	0.296	-0.41	0.182	-0.34	0.278	-0.46	0.133	-0.48	0.117	***-0.59***	***0.042***
	N (%)	-0.06	0.842	-0.05	0.888	-0.04	0.900	0.03	0.931	0.06	0.856	0.08	0.794
	**C (%)**	***-0.67***	***0.017***	***-0.67***	***0.017***	***-0.66***	***0.020***	***-0.71***	***0.009***	***-0.77***	***0.003***	***-0.76***	***0.004***
	Sugar (%)	-0.53	0.076	-0.52	0.086	-0.48	0.116	-0.52	0.081	-0.47	0.122	-0.52	0.082
	Starch (%)	-0.35	0.264	-0.39	0.206	-0.30	0.337	-0.43	0.159	-0.46	0.131	-0.53	0.075
	C: N	0.02	0.956	0.01	0.986	0.02	0.955	-0.06	0.848	-0.12	0.717	-0.13	0.683
	**Lignin: N**	-0.33	0.289	-0.38	0.217	-0.38	0.221	-0.48	0.117	-0.49	0.102	***-0.58***	***0.046***
	Ash (g m^{-2})	-0.11	0.727	-0.06	0.852	-0.05	0.887	-0.03	0.935	-0.14	0.656	-0.09	0.789
	Lignin (g m^{-2})	-0.59	**0.043**	-0.59	**0.045**	-0.57	0.055	-0.61	**0.034**	-0.68	**0.014**	-0.70	**0.011**
	Phenolics (g m^{-2})	-0.25	0.437	-0.30	0.337	-0.27	0.390	-0.35	0.262	-0.44	0.149	-0.50	0.099
	Tannin (g m^{-2})	-0.40	0.199	-0.46	0.133	-0.41	0.188	-0.51	0.090	-0.55	0.061	-0.66	**0.021**
	N (g m^{-2})	-0.46	0.129	-0.42	0.169	-0.42	0.169	-0.40	0.200	-0.43	0.161	-0.38	0.221
	C (g m^{-2})	-0.40	0.194	-0.37	0.243	-0.35	0.265	-0.38	0.225	-0.48	0.117	-0.44	0.149
	Sugar (g m^{-2})	0.46	0.131	0.46	0.129	0.41	0.187	0.46	0.132	0.33	0.297	0.38	0.223
	Starch (g m^{-2})	-0.13	0.680	-0.07	0.839	-0.11	0.742	-0.03	0.916	-0.12	0.718	-0.05	0.889
Forest gap													
	Toughness (N mm^{-1})	***-0.77***	***0.019***	***-0.82***	***0.001***	***-0.80***	***0.002***	***-0.74***	***0.006***	***-0.61***	***0.033***	***-0.66***	***0.021***
	LMA (%)	***-0.84***	***0.005***	***-0.91***	***0.000***	***-0.83***	***0.001***	***-0.62***	***0.031***	-0.44	0.150	-0.45	0.143
	Ash (%)	**0.74**	**0.026**	**0.64**	**0.025**	**0.78**	**0.003**	**0.64**	**0.024**	0.57	0.052	0.47	0.119
	Lignin (%)	-0.55	0.125	-0.49	0.108	***-0.59***	***0.042***	***-0.84***	***0.001***	***-0.92***	***0.000***	***-0.83***	***0.001***
	Phenolics (%)	-0.31	0.385	-0.23	0.463	-0.33	0.289	-0.44	0.157	-0.44	0.147	-0.39	0.213
	Tannin (%)	-0.36	0.316	-0.29	0.352	-0.39	0.216	-0.55	0.061	-0.58	0.050	-0.57	0.051
	N (%)	0.22	0.501	0.20	0.533	0.20	0.525	0.24	0.445	0.25	0.441	0.27	0.397
	C (%)	***-0.89***	***0.002***	***-0.87***	***0.000***	***-0.92***	***0.000***	***-0.85***	***0.001***	***-0.73***	***0.007***	***-0.74***	***0.006***
	Sugar (%)	-0.57	0.125	***-0.59***	***0.042***	***-0.62***	***0.033***	-0.54	0.069	-0.54	0.069	-0.47	0.123
	Starch (%)	-0.64	0.073	***-0.59***	***0.044***	***-0.63***	***0.027***	***-0.68***	***0.014***	***-0.71***	***0.010***	***-0.60***	***0.039***
	C: N	-0.36	0.317	-0.34	0.277	-0.35	0.258	-0.31	0.330	-0.28	0.385	-0.30	0.342
	Lignin: N	-0.50	0.168	-0.45	0.139	-0.52	0.082	***-0.65***	***0.021***	***-0.69***	***0.013***	***-0.64***	***0.025***
	Ash (g m^{-2})	-0.57	0.054			-0.50	0.095	-0.36	0.254	-0.20	0.543	-0.25	0.439
	Lignin (g m^{-2})	-0.86	**0.000**	-0.90	**0.000**	-0.88	**0.000**	-0.81	**0.001**	-0.70	**0.011**	-0.67	**0.016**
	Phenolics (g m^{-2})	-0.54	0.072	-0.52	0.083	-0.55	0.066	-0.56	0.057	-0.50	0.098	-0.44	0.151
	Tannin (g m^{-2})	-0.63	**0.027**	-0.62	**0.031**	-0.65	**0.022**	-0.70	**0.012**	-0.64	**0.025**	-0.62	**0.032**
	N (g m^{-2})	-0.60	**0.041**	-0.62	**0.032**	-0.59	**0.043**	-0.46	0.135	-0.34	0.274	-0.30	0.350
	C (g m^{-2})	-0.85	**0.000**	-0.92	**0.000**	-0.85	**0.001**	-0.64	**0.025**	-0.47	0.128	-0.47	0.120
	Sugar (g m^{-2})	0.09	0.783	0.05	0.886	0.15	0.633	0.22	0.488	0.36	0.252	0.24	0.452
	Starch (g m^{-2})	-0.30	0.336	-0.46	0.132	-0.30	0.349	-0.12	0.720	0.06	0.850	-0.05	0.867

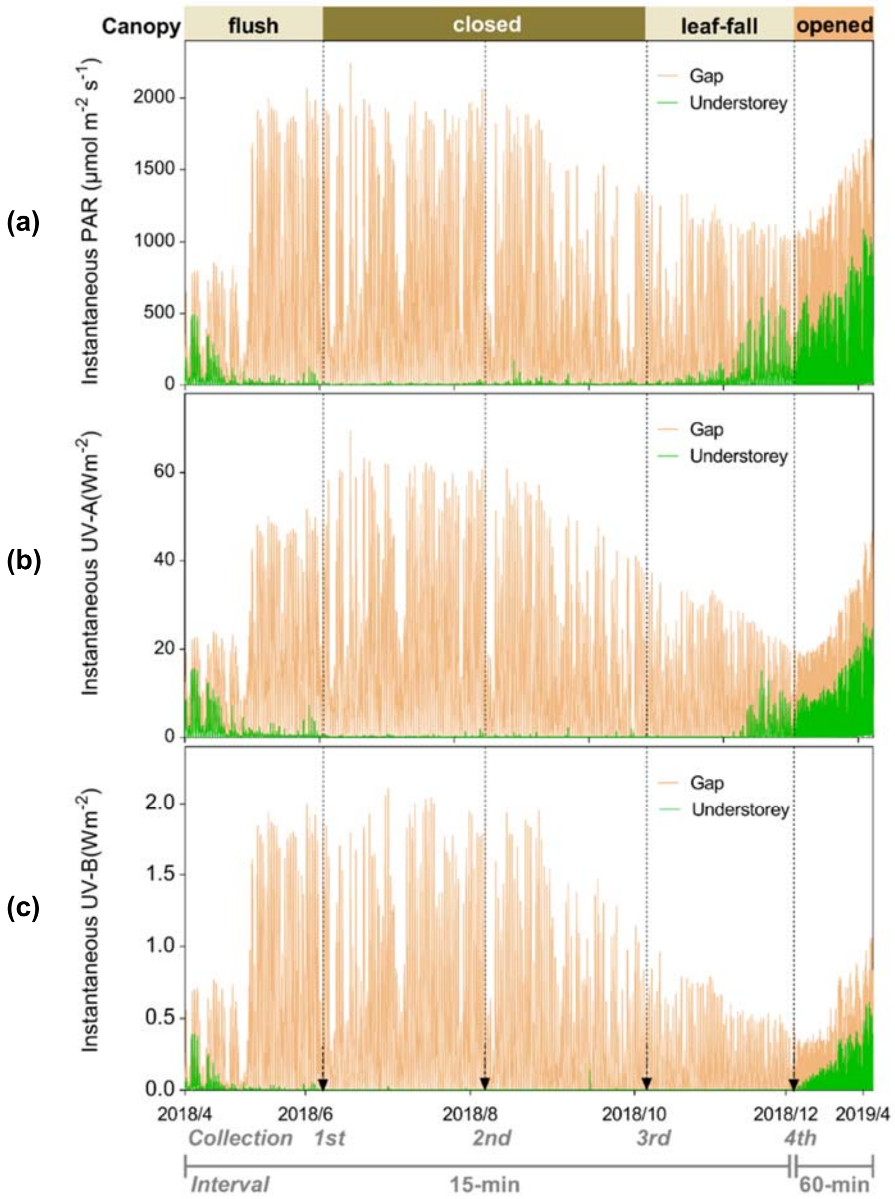

Fig. S1 Spectral irradiance in the temperate deciduous forest understorey vs. the gap was dramatically modified by canopy openness during the year. (**a**) Photosynthetically active radiation (PAR), (**b**) UV-A radiation, (**c**) UV-B radiation in the forest understorey and gap during the whole year covering the period of the field decomposition experiment in a temperate forest in central Japan (36°56'N, 140°35'E). PAR, UV-A, and UV-B radiation were concurrently measured at 15-min intervals in the experimental period using a quantum sensor (LI-190SA; LI-COR, Lincoln, NE), and two UV-Cosine sensors (UV-B and UV-A) (sglux GmbH, D-12489 Berlin, Germany), respectively, with a data-logger (LI-1400; LI-COR). Arrows denote the date of litterbox collections.

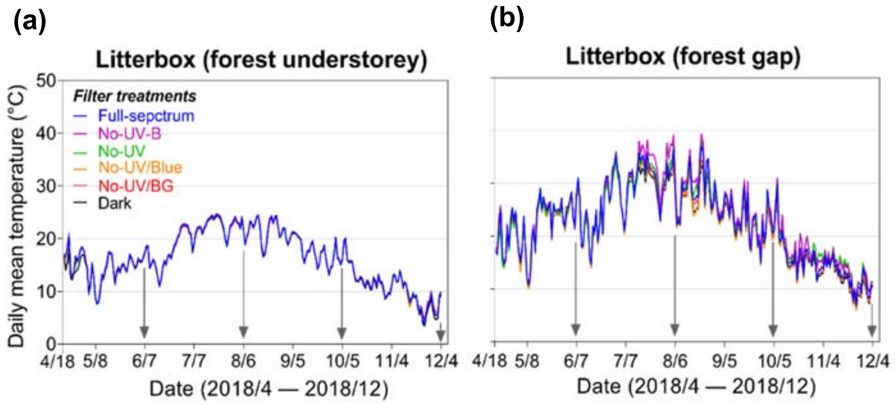

Fig. S2 Temperature in litterboxes varied greatly depending on canopy openness rather than filter treatments along the growing season in a temperate deciduous forest. Daily mean temperature in litterboxes in the forest understorey (**a**) and gap (**b**) in a temperate forest in central Japan (36°56'N, 140°35'E). Arrows denote the date of litterboxes collection. The temperature was recorded at 30-min intervals using a HOBO H8 Pro temperature logger (Onset Computer Corporation, Bourne, Massachusetts, USA), which was placed under the litter in each type of litterbox.

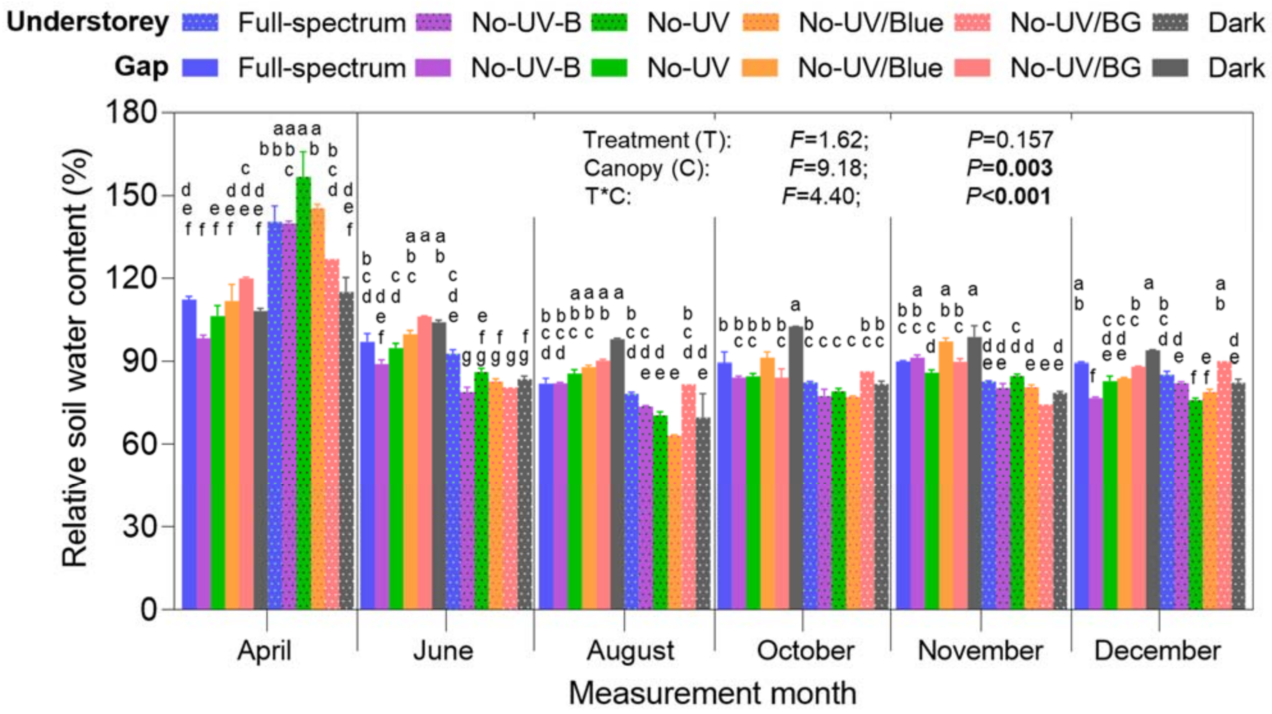

Fig. S3 Soil moisture under litterboxes across the experimental period is similar among treatments irrespective of canopy openness in the studied temperate deciduous forest. Soil samples (0-5 cm depth) under each type of filter litterbox were collected at solar noon (mean ± SEM, n = 3) at the studied plot, and relative gravimetric soil water content was calculated after drying in a muffle oven (105°C, 72 hours).

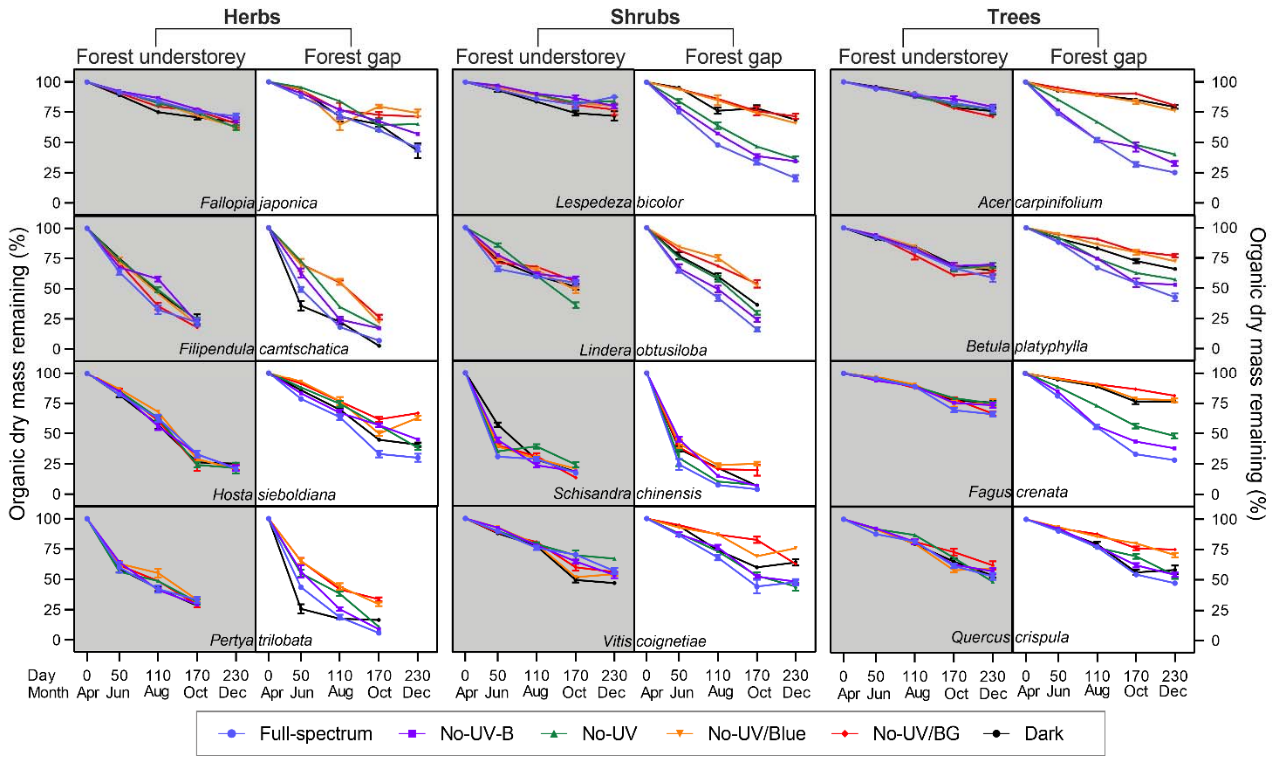

Fig. S4 Organic mass loss of 12 litter species varied significantly depending on canopy openness, filter treatment, and functional groups over eight months (April – December, 2018). Grey and white panels denote the decomposition pattern in the understorey and gap, respectively in the studied area. Filter treatments include: Full-spectrum, >280 nm, transmitting approximately 95% of the whole solar spectrum; No-UV-B, >315 nm, attenuating UV-B radiation; No-UV, >400 nm, attenuating all UV radiation; No-UV/blue, >500 nm, attenuating all UV and blue wavelengths; No-UV/BG, >580 nm, attenuating all UV radiation and blue–green (BG) wavelengths; Dark, attenuating all sunlight. Values are means (±SEM, n=4). Two herb species (*Filipendula camtschatica* and *Pertya trilobata*) and two shrub species (*Lindera obtusiloba* Blume and *Schisandra chinensis*) were totally collected at the third time (October), due to their fast decomposition rate.

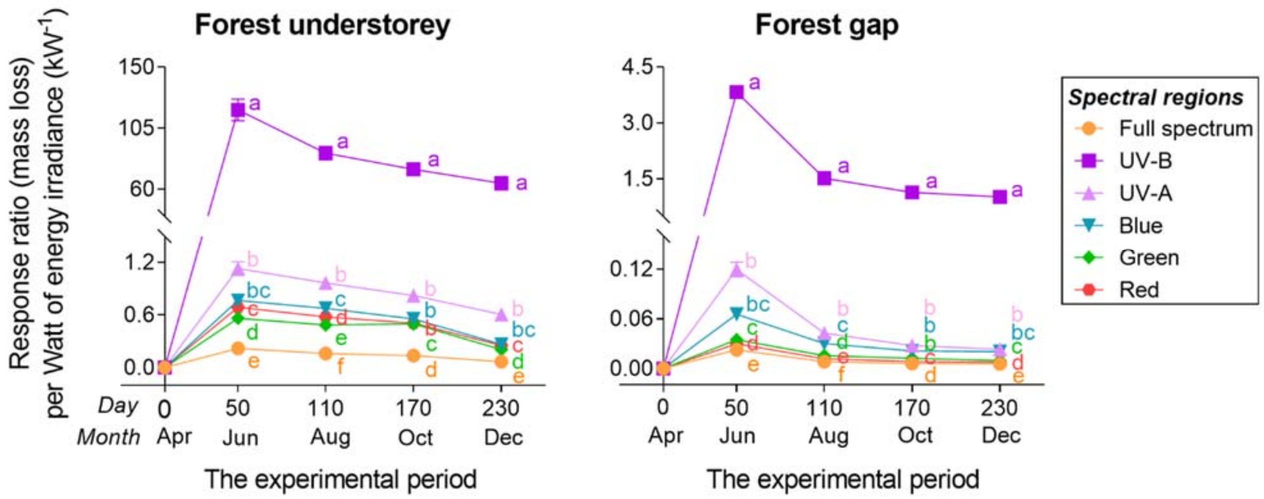

Fig. S5 Mass loss per Watt of the energy irradiance litter receives has greater response to spectral regions in the forest understorey than the gap, particularly to UV-B radiation. Response ratio of mass loss, *RR*(mass loss), to spectral regions (full spectrum, UV-B, UV-A, Blue, green, red), calculated as the ratio of mass loss between pairs of spectral treatments over eight-month decomposition, as shown in Fig. **2c**. Here *RR*(mass loss) data was express at the unit per Watt of the energy irradiance litter received at each experimental period. Different lowercase letters denote significant difference among spectral regions within a collection ($P < 0.05$), test by LSD multiple comparisons with BH's adjustment. Values are means across species (± SEM); n=12 for the first three collections, and n=8 for the final collection.

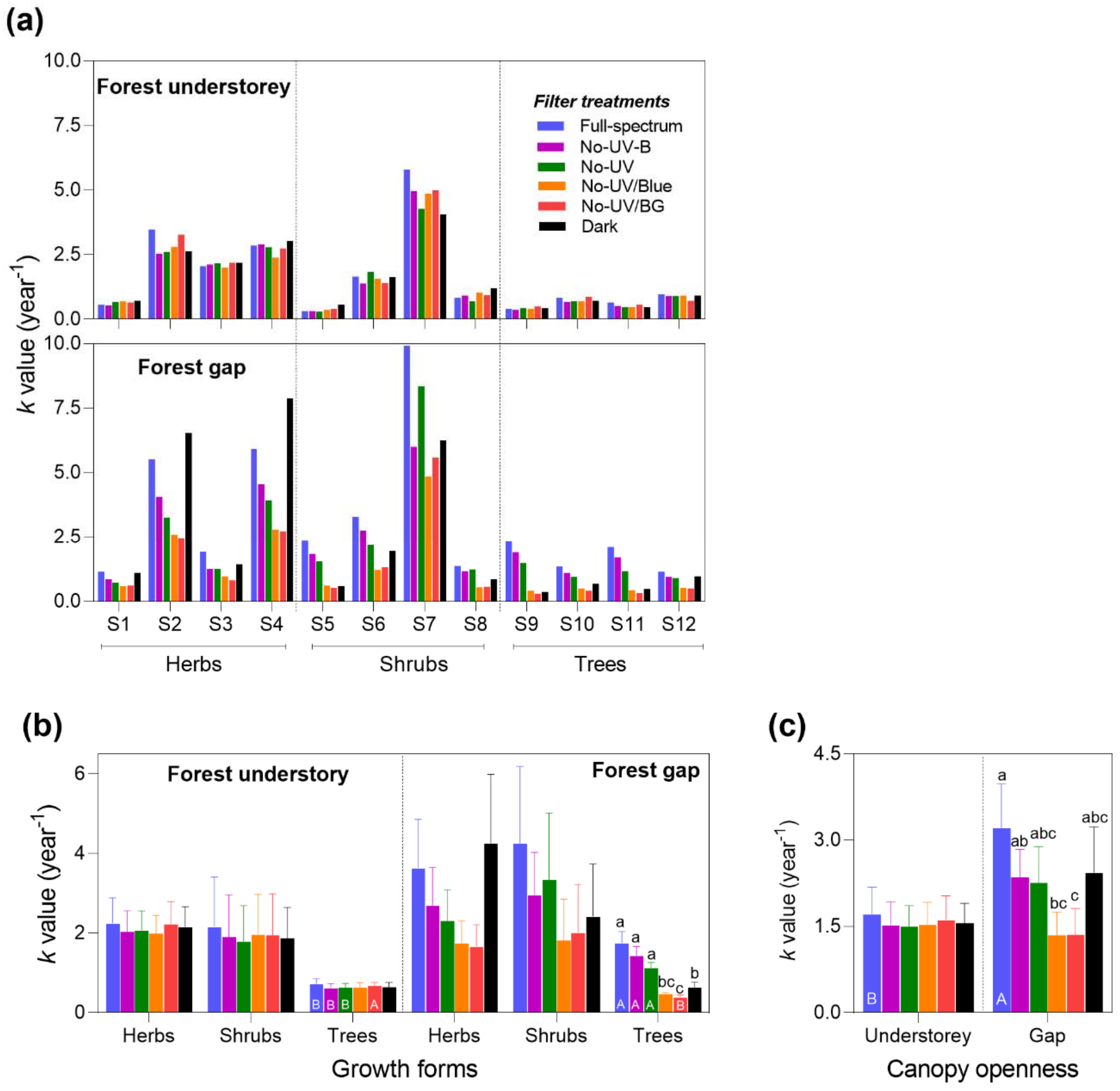

Fig. S6 Growth forms interacting with canopy openness modify litter decay rates in response to filter treatments. *k* values in leaf litter decomposition in response to filter treatments at (**a**) species level, (**b**) Growth form level (mean ± SEM, n = 4), and (**c**) plot level (mean ± SEM, n = 12) after eight-month decomposition in the studied temperate forest. Different lowercase letters in (**b**) and (**c**) denote significant difference (P <0.05) among filter treatments within each growth form and within a plot, and different uppercase letters denote significant difference between plots under the same spectral treatment.

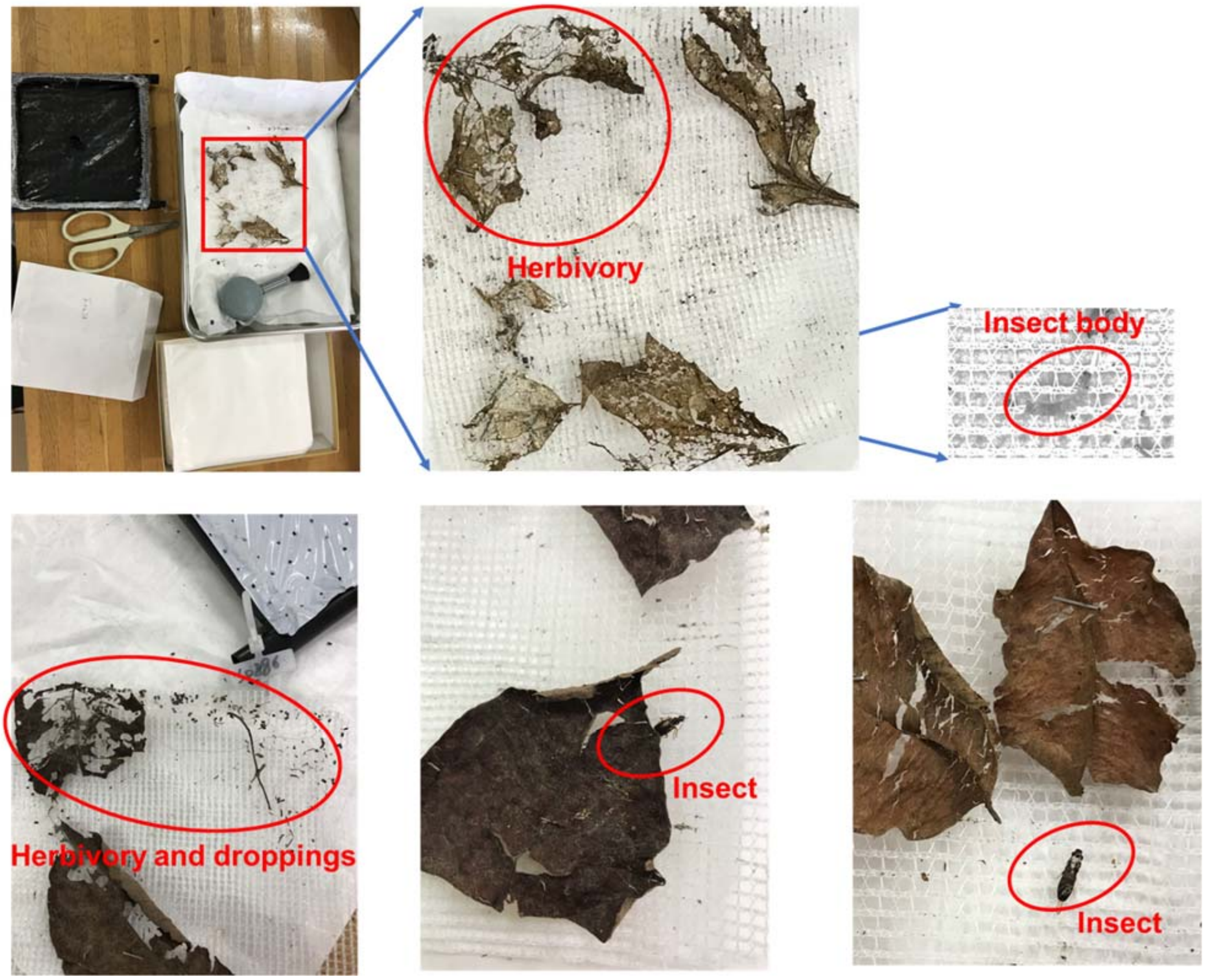

Fig. S7 Herbivory may explain the higher litter decay rate in Dark treatment than No-UV/Blue and No-UV/BG treatments those exposure to long-wavelength visible light. Photographs show the herbivory damage, insect body, and droppings on litter from Dark treatment at the third collection.

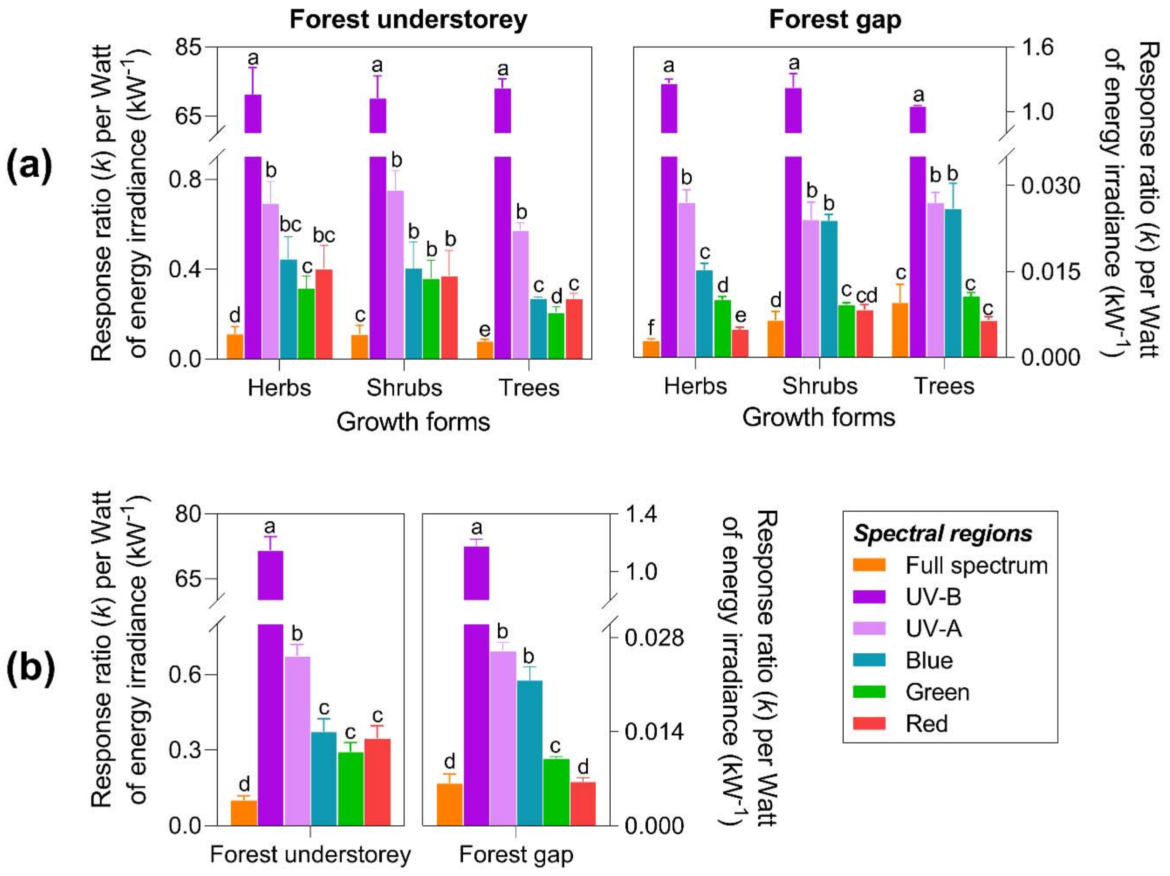

Fig. S8 Shorter-waveband spectral regions per Watt of the energy irradiance litter receives, particularly UV-B radiation, have greater importance than longer-waveband regions, modified by canopy openness and plant growth forms. (**a**) Response ratio of *k* values, *RR*(*k*) per Watt of the energy irradiance to spectral regions, calculated as the ratio of *k* values between pairs of spectral treatments divided by the irradiance litter received over the experimental period at the functional-group level (mean ±SEM, n=4). (**b**) *RR*(*k*) per Watt of the energy irradiance at the plot level (mean ±SEM, n=12). Different lowercase letters denote significant difference among spectral regions within a growth form and plot ($P < 0.05$). Difference between the understorey and gap for each spectral region was all significant ($P < 0.05$) at the functional-group and plot level, not shown on the figure.

Table S1 Initial traits of freshly senescent leaves among 12 species. Values are means (SEM) for each species (n = 5) and each growth form (n = 4). Different letters across a column are significantly different (P < 0.05) among species and growth forms, tested by Tukey's multiple comparison. Lower-case letters, significant differences among species; capital letters, significant differences among growth forms.

Growth forms	No.	Species	LMA (gm^{-2})	Toughness ($N\ mm^{-1}$)	Ash (%)	Lignin (%)	Phenolics (%)	Tannin (%)	N (%)	C (%)	Sugar (%)	Starch (%)	C: N	Lignin: N
Herbs	S1	*Pertya trilobata*	33.7(0.69)f	0.6(0.01)f	5.8(0.13)cd	11.5(0.10)d	0.6(0.09)g	0.3(0.02)f	1.5(0.1)c	42.8(0.15)f	0.5(0.01)d	6.5(0.24)bc	29.1(0.49)bc	8.6(0.18)c
	S2	*Filipendula camtschatica*	35.1(0.49)f	0.6(0.03)f	8.1(0.12)e	12.4(0.35)cd	4.4(0.16)d	0.3(0.01)f	1.7(0.4)b	42.8(0.26)f	0.6(0.02)cd	5.7(0.27)bcd	25.2(0.31)a	7.3(0.24)c
	S3	*Hosta sieboldiana*	63.5(1.15)c	1.7(0.10)cd	6.2(0.20)d	11.3(0.12)bc	0.3(0.04)h	0.2(0.02)f	0.7(0.1)e	45.8(0.76)cde	0.3(0.02)efg	4.7(0.39)cde	63.9(1.60)f	15.8(0.53)de
	S4	*Fallopia japonica*	95.3(2.40)a	3.2(0.35)a	5.2(0.16)b	16.9(0.29)ef	2.7(0.10)e	0.8(0.05)d	1.9(0.3)a	48.1(0.47)ab	0.2(0.01)h	5.9(0.98)bcd	24.7(0.43)a	8.1(0.13)ef
Shrubs	S5	*Schisandra chinensis*	24.8(0.43)h	0.7(0.06)f	12.4(0.53)f	7.8(0.11)a	1.6(0.09)f	0.4(0.03)e	1.5(0.1)c	40.5(0.12)g	1.7(0.06)b	14.8(0.67)a	27.8(0.12)b	6.6(0.07)bc
	S6	*Lindera obtusiloba Blume*	31.6(0.78)g	1.2(0.15)e	7.3(0.11)e	11.3(0.19)b	7.8(0.16)b	2.0(0.06)a	0.7(0.2)e	45.4(0.26)de	2.9(0.07)a	5.3(0.09)bcde	61.8(3.31)f	15.2(0.85)a
	S7	*Lespedeza bicolor Turcz. var. bicolor.*	44.1(0.95)e	2.2(0.26)bc	5.2(0.05)bc	16.6(0.16)f	1.8(0.05)f	0.6(0.01)e	1.4(0.2)c	46.8(0.30)bcd	0.4(0.00)defg	6.9(0.33)b	32.7(0.91)d	11.5(0.22)cd
	S8	*Vitis coignetiae*	70.6(1.82)b	1.3(0.08)de	7.5(0.13)e	12.6(0.61)e	1.6(0.08)f	0.4(0.03)e	1.5(0.6)c	45.1(0.22)de	0.4(0.04)def	5.5(0.42)bcde	30.6(0.57)cd	9.9(0.52)b
Trees	S9	*Acer carpinifolium*	42.3(1.00)e	1.3(0.04)de	8.0(0.15)e	21.3(0.59)g	5.4(0.28)c	1.5(0.10)bc	1.4(0.6)c	44.8(0.31)e	0.3(0.01)fgh	3.7(0.31)e	32.4(0.65)d	15.6(0.36)g
	S10	*Fagus crenata Blume*	50.3(0.92)d	2.8(0.15)ab	3.9(0.06)a	23.0(0.26)g	8.5(0.34)ab	1.8(0.03)ab	0.9(0.3)e	48.4(0.22)ab	0.5(0.02)de	5.0(0.49)bcde	57.1(2.40)f	24.8(0.90)f
	S11	*Betula platyphylla*	72.9(1.11)b	2.6(0.11)ab	4.8(0.12)b	16.4(0.06)f	5.1(0.27)cd	1.7(0.13)ab	1.1(0.1)d	48.7(0.46)a	0.3(0.02)gh	4.3(0.33)de	45.8(0.64)e	15.3(0.29)h
	S12	*Quercus crispula Blume*	74.9(1.80)b	2.8(0.13)ab	6.2(0.22)d	15.7(0.38)f	9.5(0.30)a	1.3(0.05)c	0.8(0.4)e	47.4(0.41)abc	1.0(0.17)c	3.6(0.38)e	58.3(1.93)f	20.0(0.50)fg
		Herb	57.5(5.68)A	1.5(0.26)B	6.3(0.26)AB	13.0(0.40)B	2.0(0.39)B	0.4(0.05)C	1.5(0.1)A	44.9(0.55)B	0.4(0.04)B	5.7(0.30)B	35.8(3.77)B	10.0(0.79)B
		Shrub	42.5(4.20)B	1.4(0.14)B	8.1(0.61)A	12.9(0.63)B	3.2(0.62)B	0.9(0.15)B	1.3(0.1)AB	44.5(0.55)B	1.4(0.24)A	8.1(0.91)A	38.2(3.25)B	10.8(0.75)B
		Tree	60.9(3.36)A	2.4(0.15)A	5.7(0.36)B	18.8(0.60)A	7.2(0.46)A	1.6(0.06)A	1.0(0.1)B	47.3(0.39)A	0.5(0.08)B	4.2(0.21)C	48.4(2.51)A	18.9(0.92)A

Table S2 The proportion of solar radiation transmitted as spectral photon irradiance, and spectral photon ratios, under each type of filter employed in the experiment for the regions of the spectrum of interest measured, at solar noon on 23-07-2018.

Quantity	Ambient	Full-spectrum	No UV-B	No UV	No UV/Blue	No UV/BG	Dark
**Transmittance**							
PAR	1.00	0.88	0.83	0.84	0.63	0.35	0.02
UV-B	1.00	0.85	0.00	0.03	0.00	0.00	0.00
UV-A	1.00	0.88	0.76	0.06	0.02	0.01	0.00
Blue	1.00	0.89	0.84	0.86	0.03	0.01	0.01
Green	1.00	0.88	0.84	0.85	0.74	0.02	0.02
Red	1.00	0.87	0.83	0.84	0.91	0.82	0.03
Far_red	1.00	0.87	0.83	0.83	0.91	0.88	0.04
**Photon ratios (mol mol^{-1})**							
Blue: PAR	0.30	0.30	0.30	0.30	0.01	0.01	0.25
Green: PAR	0.37	0.37	0.37	0.37	0.46	0.03	0.35
Red: PAR	0.33	0.33	0.33	0.33	0.52	0.96	0.40

Table S3 Linear Mixed-Effects Models (LMM) results for three fixed factors (time, canopy, and filter treatment) and their interactions on litter mass loss, with species and blocks as the random factors. Block was dropped in the analysis, because it did not have significant effect on mass loss, but made the model singularity. *P*-values in bold indicate statistically significant differences.

Factor	DF	*F*	*P*
Time (T)	1	4802.3	**<0.001**
Canopy (C)	1	284.1	**<0.001**
Filter treatment (FT)	5	108.2	**<0.001**
T×C	1	0.8	0.366
T×FT	5	1.8	0.111
C×FT	5	104.9	**<0.001**
T×C×FT	5	5.4	**<0.001**

Table S4 The effect of individual spectral regions on litter mass loss calculated through pairwise comparisons of filter treatments. Grey shading denotes the contrasts among individual spectral regions (rather than broader combinations of spectral regions) that were the main focus of this study. For contrasts α = 0.05 was used. *P*-values in bold indicate statistically significant differences.

**Effect of…**	**Contrasts**			**Decomposition period**															
				**50 days**				**110 days**				**170 days**				**230 days**			
	Treatment comparison			Estimate	SE	z	*P*	Estimate	SE	z	*P*	Estimate	SE	z	*P*	Estimate	SE	z	*P*
Forest understorey (one-way ANOVA results)				***(df = 5, F= 7.5, P < 0.001)***				***(df = 5, F= 6.8, P < 0.001)***				***(df = 5, F= 3.7, P = 0.003)***				*(df = 5, F= 1.5, P = 0.199)*			
Full spectrum	Full-spectrum	vs.	Dark	**3.88**	**0.80**	**4.87**	**<0.001**	0.59	1.01	0.58	0.992	-2.55	1.08	-2.36	0.171	-1.48	1.47	-1.01	0.916
UV-B	Full-spectrum	vs.	No-UV-B	**4.22**	**0.80**	**5.26**	**<0.001**	2.45	1.00	2.44	0.143	0.97	1.10	0.88	0.951	1.41	1.47	0.96	0.931
UV-A	No-UV-B	vs.	No-UV	-0.38	0.80	-0.47	0.997	1.76	1.00	1.76	0.491	-1.58	1.11	-1.43	0.711	0.11	1.48	0.07	1.000
Blue	No-UV	vs.	No-UV/Blue	0.28	0.80	0.36	0.999	0.27	0.99	0.27	1.000	-1.56	1.10	-1.42	0.715	-0.82	1.49	-0.55	0.994
Green	No-UV/Blue	vs.	No-UV/BG	-1.25	0.80	-1.56	0.623	-2.61	0.98	-2.65	0.085	0.42	1.09	0.39	0.999	-1.85	1.51	-1.23	0.824
Red	No-UV/BG	vs.	Dark	1.00	0.80	1.26	0.809	-1.27	1.00	-1.27	0.799	-0.81	1.08	-0.75	0.976	-0.34	1.49	-0.22	1.000
UV	Full-spectrum	vs.	No-UV	**3.84**	**0.80**	**4.82**	**<0.001**	**4.20**	**1.01**	**4.15**	**<0.001**	-0.61	1.10	-0.55	0.994	1.52	1.47	1.03	0.907
UV/Blue	Full-spectrum	vs.	No-UV/Blue	**4.13**	**0.80**	**5.17**	**<0.001**	**4.47**	**1.00**	**4.48**	**<0.001**	-2.16	1.09	-1.98	0.351	0.70	1.48	0.47	0.997
UV/BG	Full-spectrum	vs.	No-UV/BG	**2.88**	**0.80**	**3.61**	**0.004**	1.86	1.00	1.85	0.430	-1.74	1.09	-1.60	0.600	-1.15	1.48	-0.77	0.972
UV-A/Blue	No-UV-B	vs.	No-UV/Blue	-0.09	0.80	-0.12	1.000	2.03	0.98	2.06	0.310	**-3.13**	**1.10**	**-2.85**	**0.050**	-0.71	1.49	-0.47	0.997
UV-A/BG	No-UV-B	vs.	No-UV/BG	-1.34	0.80	-1.67	0.551	-0.58	0.99	-0.59	0.992	-2.71	1.10	-2.46	0.135	-2.56	1.49	-1.71	0.525
UV-A/BGR	No-UV-B	vs.	Dark	-0.34	0.80	-0.42	0.998	-1.86	1.00	-1.86	0.427	**-3.52**	**1.09**	**-3.22**	**0.016**	-2.89	1.48	-1.95	0.371
BG	No-UV	vs.	No-UV/BG	-0.96	0.80	-1.21	0.833	-2.34	1.00	-2.35	0.175	-1.14	1.10	-1.04	0.906	-2.66	1.49	-1.78	0.477
BGR	No-UV	vs.	Dark	0.04	0.80	0.05	1.000	**-3.62**	**1.01**	**-3.59**	**0.004**	-1.94	1.09	-1.79	0.473	-3.00	1.48	-2.02	0.328
GR	No-UV/Blue	vs.	Dark	-0.25	0.80	-0.31	1.000	**-3.88**	**0.99**	**-3.91**	**0.001**	-0.39	1.08	-0.36	0.999	-2.18	1.49	-1.46	0.689
Forest gap (one-way ANOVA results)				***(df = 5, F= 36.3, P < 0.001)***				***(df = 5, F= 84.9, P < 0.001)***				***(df = 5, F= 100.8, P = 0.003)***				***(df = 5, F= 92.4, P = 0.199)***			
Full spectrum	Full-spectrum	vs.	Dark	**5.87**	**1.41**	**4.15**	**<0.001**	**14.17**	**1.51**	**9.38**	**<0.001**	**17.09**	**1.60**	**10.66**	**<0.001**	**26.17**	**2.23**	**11.74**	**<0.001**
UV-B	Full-spectrum	vs.	No-UV-B	**5.73**	**1.41**	**4.07**	**0.001**	**5.53**	**1.46**	**3.78**	**0.002**	**8.36**	**1.61**	**5.20**	**<0.001**	**9.33**	**2.21**	**4.22**	**<0.001**
UV-A	No-UV-B	vs.	No-UV	2.82	1.41	2.00	0.340	**6.32**	**1.46**	**4.32**	**<0.001**	3.54	1.60	2.21	0.231	2.75	2.21	1.24	0.816
Blue	No-UV	vs.	No-UV/Blue	**5.24**	**1.42**	**3.69**	**0.003**	**11.10**	**1.49**	**7.44**	**<0.001**	**16.60**	**1.59**	**10.47**	**<0.001**	**23.95**	**2.23**	**10.75**	**<0.001**
Green	No-UV/Blue	vs.	No-UV/BG	-0.19	1.42	-0.13	1.000	0.97	1.51	0.64	0.988	2.59	1.61	1.61	0.593	1.37	2.23	0.61	0.990
Red	No-UV/BG	vs.	Dark	**-7.73**	**1.41**	**-5.47**	**<0.001**	**-9.76**	**1.52**	**-6.41**	**<0.001**	**-14.00**	**1.62**	**-8.65**	**<0.001**	**-11.23**	**2.23**	**-5.04**	**<0.001**
UV	Full-spectrum	vs.	No-UV	**8.55**	**1.41**	**6.05**	**<0.001**	**11.85**	**1.46**	**8.14**	**<0.001**	**11.90**	**1.59**	**7.48**	**<0.001**	**12.08**	**2.23**	**5.42**	**<0.001**
UV/Blue	Full-spectrum	vs.	No-UV/Blue	**13.80**	**1.42**	**9.70**	**<0.001**	**22.95**	**1.49**	**15.38**	**<0.001**	**28.51**	**1.59**	**17.90**	**<0.001**	**36.03**	**2.23**	**16.17**	**<0.001**
UV/BG	Full-spectrum	vs.	No-UV/BG	**13.60**	**1.41**	**9.62**	**<0.001**	**23.92**	**1.47**	**16.27**	**<0.001**	**31.09**	**1.62**	**19.25**	**<0.001**	**37.40**	**2.23**	**16.78**	**<0.001**
UV-A/Blue	No-UV-B	vs.	No-UV/Blue	**8.06**	**1.41**	**5.70**	**<0.001**	**17.42**	**1.50**	**11.63**	**<0.001**	**20.14**	**1.60**	**12.58**	**<0.001**	**26.70**	**2.21**	**12.07**	**<0.001**
UV-A/BG	No-UV-B	vs.	No-UV/BG	**7.87**	**1.41**	**5.59**	**<0.001**	**18.39**	**1.48**	**12.45**	**<0.001**	**22.73**	**1.62**	**14.00**	**<0.001**	**28.06**	**2.21**	**12.69**	**<0.001**
UV-A/BGR	No-UV-B	vs.	Dark	0.14	1.41	0.10	1.000	**8.63**	**1.52**	**5.70**	**<0.001**	**8.73**	**1.61**	**5.41**	**<0.001**	**16.84**	**2.21**	**7.61**	**<0.001**
BG	No-UV	vs.	No-UV/BG	**5.05**	**1.41**	**3.57**	**0.005**	**12.07**	**1.47**	**8.21**	**<0.001**	**19.19**	**1.61**	**11.93**	**<0.001**	**25.32**	**2.23**	**11.36**	**<0.001**
BGR	No-UV	vs.	Dark	-2.68	1.41	-1.90	0.404	2.32	1.51	1.53	0.642	**5.19**	**1.60**	**3.25**	**0.015**	**14.09**	**2.23**	**6.32**	**<0.001**
GR	No-UV/Blue	vs.	Dark	**-7.92**	**1.42**	**-5.57**	**<0.001**	**-8.79**	**1.54**	**-5.70**	**<0.001**	**-11.41**	**1.60**	**-7.15**	**<0.001**	**-9.86**	**2.23**	**-4.43**	**<0.001**

Table S5 Linear Mixed-Effects Models (LMM) results for three fixed factors (time, canopy, and spectral region) and their interactions affecting the response ratio of mass loss, *RR*(mass loss), calculated as the ratio of mass loss between pairs of spectral treatments, and its values per Watt of energy irradiance (kW^{-1}) litter received. Species were set as the random factor. Significant terms are shown in bold.

Factor	Df	*RR*(mass loss)		*RR*(mass loss) per Watt of energy irradiance	
		F	***P***	***F***	***P***
Canopy (C)	1	58.8	**<0.001**	9853.3	**<0.001**
Time (T)	1	2.4	0.122	682.5	**<0.001**
Spectra region (SR)	5	35.2	**<0.001**	1765.0	**<0.001**
C×T	1	2.6	0.108	141.4	**<0.001**
C×SR	5	27.1	**<0.001**	98.7	**<0.001**
T×SR	5	1.6	0.161	10.3	**<0.001**
C×T×SR	5	1.3	0.253	2.3	**0.045**

Table S6 Linear Mixed-Effects Models (LMM) results for three fixed factors (canopy, growth form and filter treatments) and their interactions on *k* values (decay constant), with species as the random factor. Significant terms are shown in bold.

Factor	DF	*F*	*P*
Canopy (C)	1	23.0	**<0.001**
Growth form (GF)	2	2.2	0.168
Filter treatment (FT)	5	9.5	**<0.001**
C×GF	2	4.4	**0.015**
C×FT	5	12.5	**<0.001**
GF×FT	10	1.7	0.085
C×GF×FT	10	2.1	**0.031**

Table S7 Linear Mixed-Effects Models (LMM) results for three fixed factors (canopy, growth form and filter treatments) and their interactions on response ratio of *k* values, *RR*(*k*), and its values per Watt of energy irradiance (kW^{-1}) litter received, with species as the random factor. Significant terms are shown in bold.

Factor	Df	*RR*(k)		*RR*(*k*) per Watt of energy irradiance	
		F	***P***	***F***	***P***
Canopy (C)	1	32.0	**<0.001**	4983.2	**<0.001**
Growth form (GF)	2	5.6	**0.027**	0.3	0.729
Spectral region (SR)	5	19.3	**<0.001**	1373.6	**<0.001**
C×GF	2	3.2	**0.045**	9.8	**<0.001**
C×SR	5	16.8	**<0.001**	21.3	**<0.001**
GF×SR	10	1.5	0.170	1.1	0.377
C×GF×SR	10	3.1	**0.002**	2.4	**0.014**

Table S8 Correlations between initial litter traits and response ratio of *k* values, *RR*(*k*) in six spectral regions. Values are coefficients of determination (*R*) and *P*-values (n = 12). Normal and Italic values denote positive and negative correlations, respectively, while the bold shading denotes significant correlations (*P* <0.05).

Plots Initial traits	***RR**full spectrum*		***RR**UV-B*		***RR**UV-A*		***RR**blue*		***RR**green*		***RR**red*	
	R	*P*	*R*	*P*	*R*	*P*	*R*	*P*	*R*	*P*	*R*	*P*
Forest understorey												
Toughness (N mm^{-1})	-0.17	0.603	-0.04	0.893	-0.18	0.568	-0.08	0.816	0.20	0.533	-0.19	0.559
LMA (%)	-0.37	0.235	-0.34	0.275	-0.10	0.747	-0.19	0.562	0.32	0.304	-0.29	0.362
Ash (%)	0.29	0.367	0.14	0.674	0.18	0.569	-0.22	0.489	0.13	0.686	0.27	0.398
Lignin (%)	-0.13	0.685	0.11	0.736	-0.09	0.786	-0.08	0.809	-0.28	0.373	0.09	0.772
Phenolics (%)	0.43	0.166	**0.63**	**0.029**	-0.32	0.313	0.18	0.578	0.24	0.450	0.11	0.732
Tannin (%)	0.27	0.392	0.52	0.083	-0.43	0.164	0.30	0.337	0.04	0.896	0.15	0.637
N (%)	-0.29	0.354	-0.17	0.597	0.05	0.876	-0.37	0.238	-0.13	0.682	0.01	0.972
C (%)	-0.24	0.449	-0.04	0.914	-0.26	0.421	0.03	0.917	0.09	0.791	-0.22	0.486
Sugar (%)	-0.43	0.160	-0.31	0.321	-0.15	0.634	-0.04	0.904	-0.40	0.199	0.07	0.835
Starch (%)	0.01	0.987	0.13	0.680	-0.36	0.251	0.27	0.391	0.06	0.860	0.00	0.989
C: N	0.23	0.467	0.14	0.662	-0.18	0.581	0.40	0.194	0.22	0.490	-0.07	0.829
Lignin: N	0.24	0.453	0.25	0.432	-0.07	0.822	0.23	0.474	0.00	1.000	0.07	0.823
Forest gap												
Toughness (N mm-1)	0.31	0.326	-0.34	0.286	0.34	0.285	0.07	0.818	0.33	0.299	0.00	0.997
LMA (%)	-0.06	0.846	-0.31	0.324	0.08	0.799	-0.23	0.474	0.13	0.689	-0.33	0.300
Ash (%)	-0.08	0.800	0.53	0.075	***-0.72***	***0.008***	-0.02	0.944	-0.55	0.065	0.39	0.216
Lignin (%)	**0.60**	**0.037**	-0.56	0.060	**0.61**	**0.035**	**0.64**	**0.025**	**0.74**	**0.006**	0.18	0.566
Phenolics (%)	0.29	0.354	***-0.58***	***0.050***	0.50	0.099	0.34	0.278	0.20	0.530	-0.08	0.815
Tannin (%)	0.55	0.062	***-0.62***	***0.031***	0.52	0.083	0.51	0.089	0.28	0.384	0.15	0.641
N (%)	-0.34	0.280	0.21	0.522	-0.10	0.758	-0.25	0.425	-0.30	0.350	0.03	0.915
C (%)	0.36	0.249	-0.57	0.055	0.55	0.066	0.17	0.592	0.48	0.113	-0.12	0.717
Sugar (%)	0.09	0.777	-0.08	0.802	0.31	0.321	-0.07	0.820	0.53	0.077	-0.13	0.687
Starch (%)	0.27	0.400	-0.54	0.073	0.39	0.208	0.29	0.353	0.57	0.055	-0.22	0.498
C: N	0.27	0.399	-0.21	0.514	0.19	0.561	0.11	0.738	0.27	0.388	-0.14	0.664
Lignin: N	0.54	0.072	-0.45	0.144	0.46	0.136	0.47	0.125	**0.62**	**0.031**	-0.01	0.982
